# Supplementary material for: Mammalian APE1 controls miRNA processing and its interactome is linked to cancer RNA metabolism
Source: Nat Commun. 2017 Oct 6;8:797. doi: 10.1038/s41467-017-00842-8 (PMC5630600; doi:10.1038/s41467-017-00842-8)
Supplement: Supplementary file 1 — Supplementary Information [file 41467_2017_842_MOESM1_ESM.pdf]

SUPPLEMENTARY INFORMATION

List of contents:

- Pages 1 to 21 , Supplementary Figures;
- Page 22, Supplementary Tables;
- Pages 23 to 28, Supplementary Notes;
- Pages 29 to 33, Supplementary Methods;
- Pages 34 to 36, Supplementary References.

Supplementary Figures

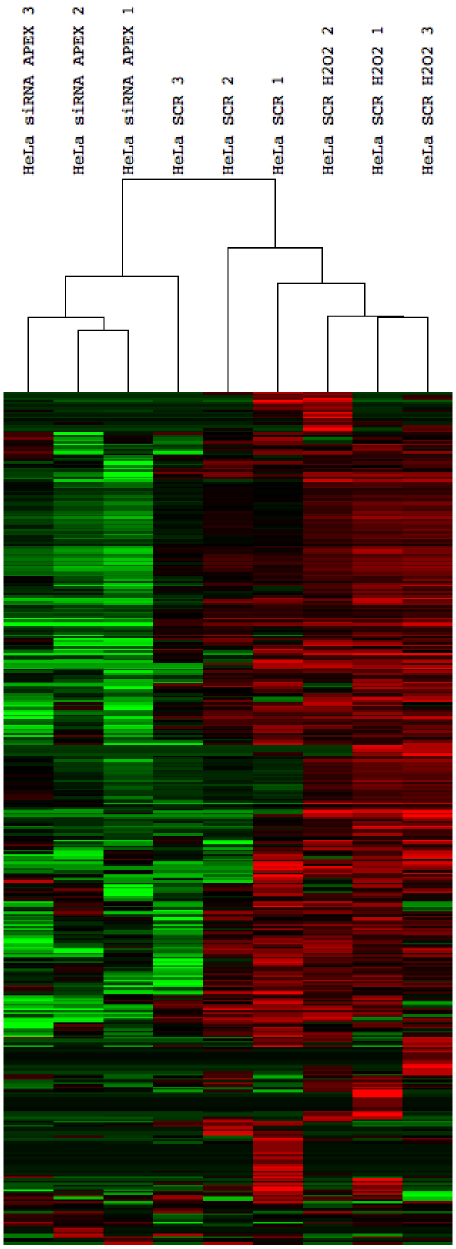

Global Clustering

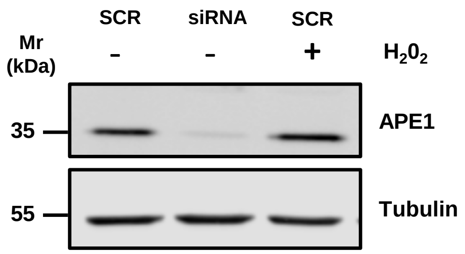

Supplementary Figure 1

a) Left panel shows hierarchical clustering of gene expression data. After normalization and log-transformation, the genes with low standard deviation were filtered (SD filter =0.2). Right panels show APE1 protein level evaluated in HeLa cells clones silenced for 10 days with doxycycline and treated with 1 mM H_20_2 for 15 min. SCR represents control clone expressing the scramble shRNA, siRNA, clones silenced for APE1. Representative Western blotting analyses of total cell extracts of HeLa cell clones probed with APE1 antibody. Tubulin was used as loading control and for data normalization.

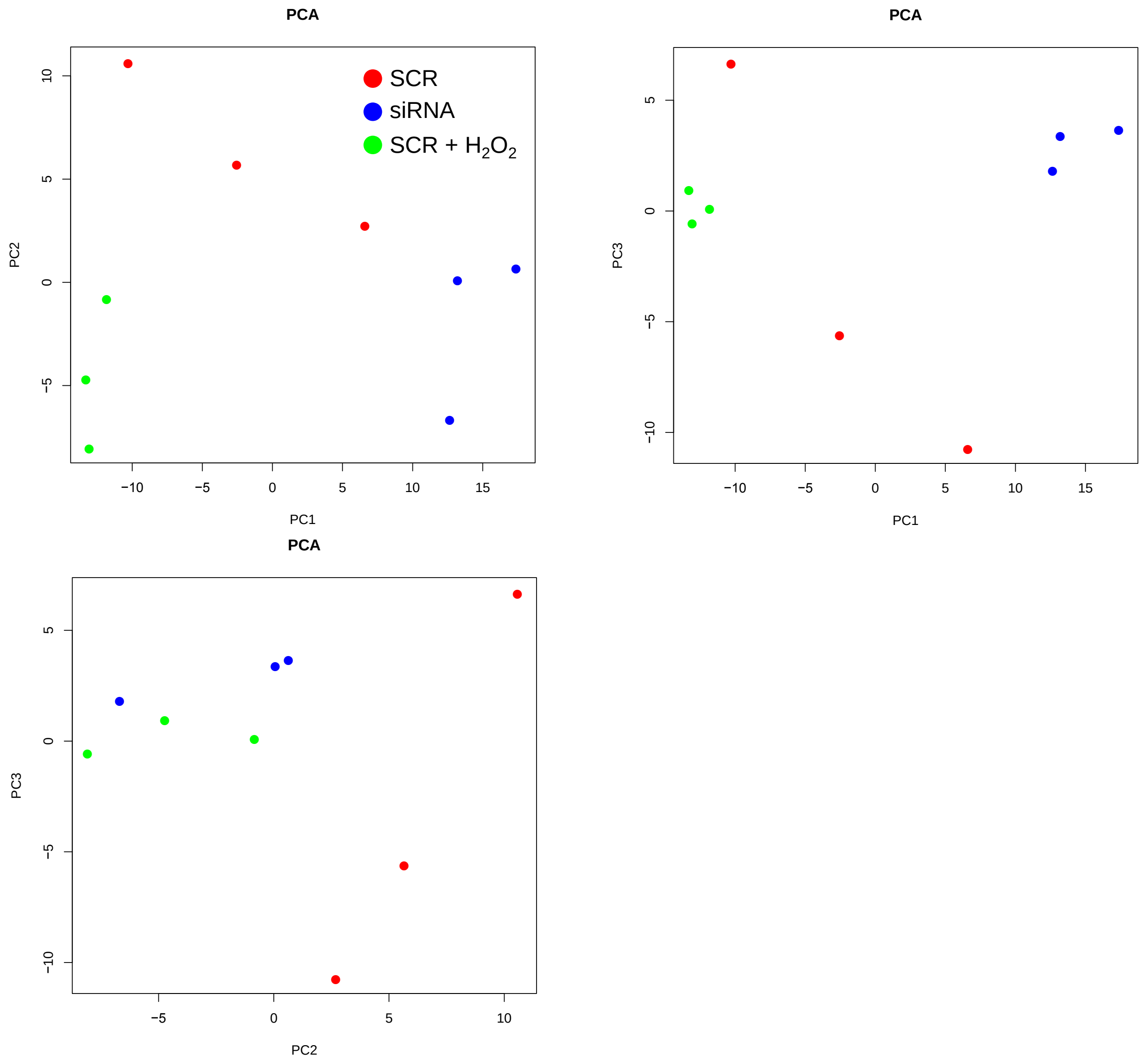

Supplementary Figure 1

b) In order to evaluate the similarities among the replicas and the different experimental conditions Principal component analysis (PCA) was performed on the nine samples miRNA expression matrix. The first three principal components are plotted in pairs and each experimental condition is highlighted by a different color. The emerging sample groups confirmed that each condition is in a separate cluster, representing in this way a homogeneous and distinct cell population.

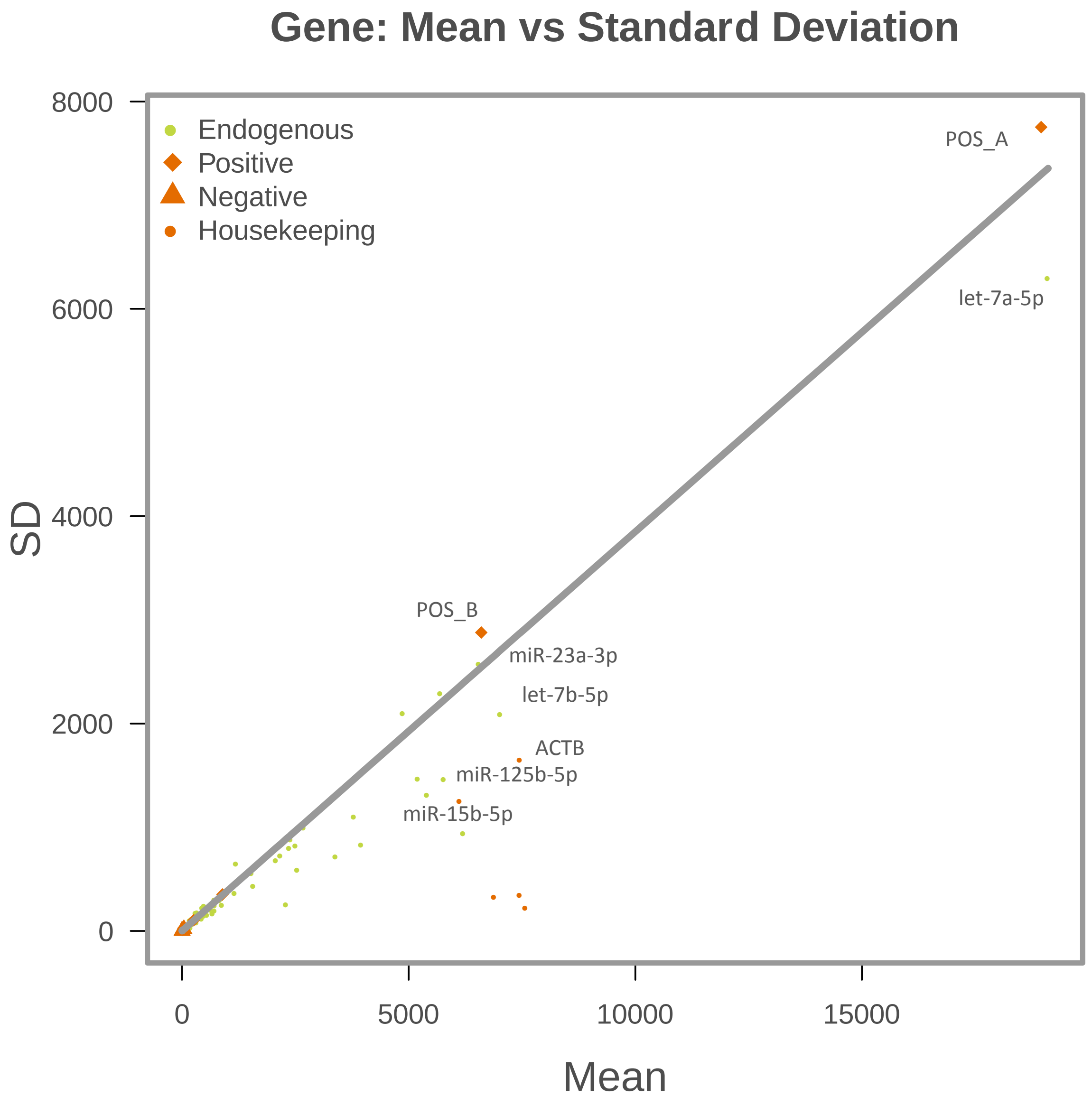

Supplementary Figure 1

c) Mean vs standard deviation plot. Green dots are endogenous genes and orange are controls, with the symbol indicating the specific type. The grey line is a best-fit loess curve through the data. As expected, the three housekeeping genes used for data normalization have high mean and low SD.

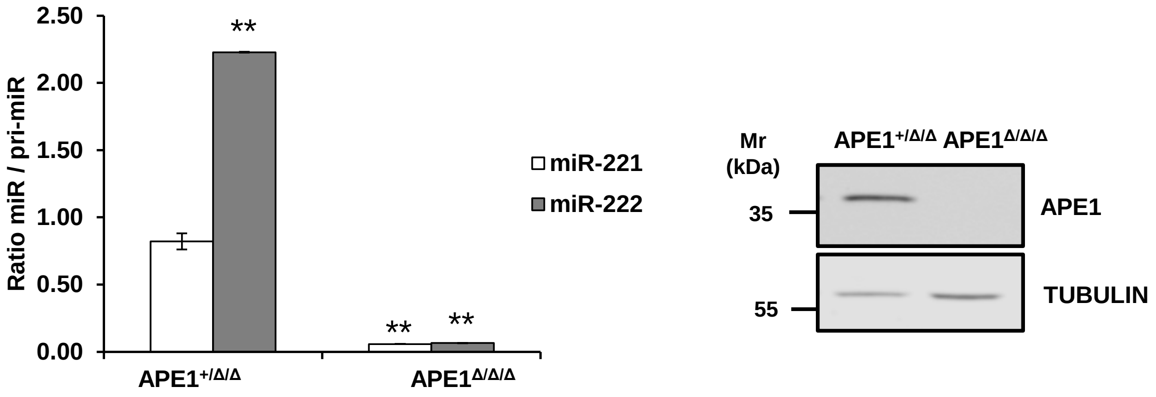

Supplementary Figure 2
Mature miR to pri-miR ratios expression levels evaluated by qRT-PCR analysis of APE1-null CH12F3 cells. Total RNA was extracted from CH12F3 APE1$^{\Delta/+/+}$ and APE1$^{\Delta/\Delta/\Delta}$ cells and reverse transcribed. Histograms show mature miR-221 and miR-222 measured by qRT-PCR analysis, normalized to RNU44, and expressed relative to GAPDH-normalized pri-miR-221/222. Asterisks represent a significant difference with respect to control (APE1 d++). ** P< 0.001.

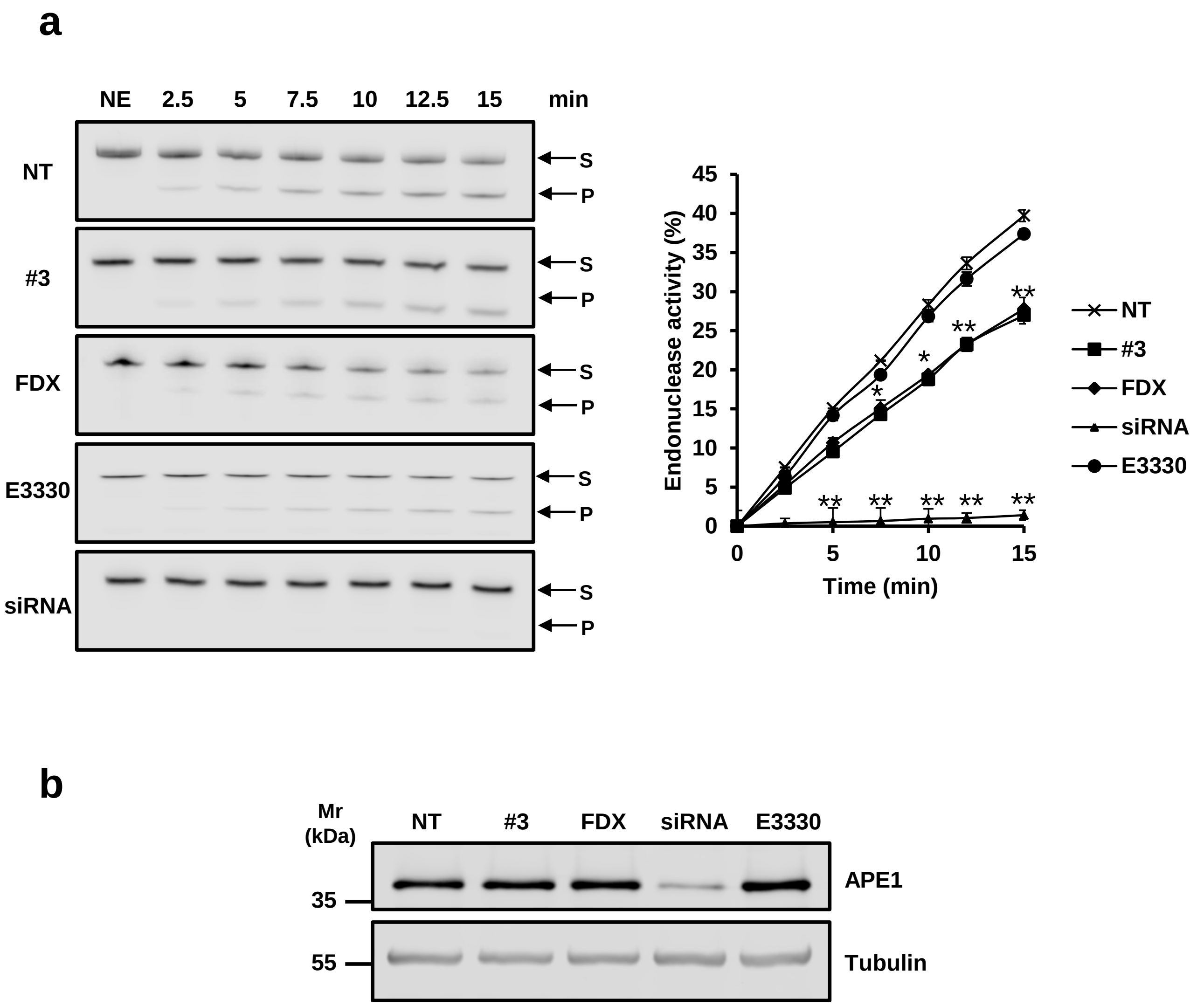

Supplementary Figure 3

a) APE1 inhibitors negatively affect AP-site incision activity by APE1. APE1 endonuclease activity was measured *in vitro* on total cell extracts from HeLa cells treated with 20 µM compound #3, 40 µM fiduxosin (FDX), 100 µM E3330 for 24 h, or HeLa cells silenced for APE1 (siRNA), as indicated in Methods section. Histogram reports time-dependent kinetics of 12.5 ng cell extracts endonuclease activity expressed as percentage conversion of an AP site-containing DNA substrate (S) to the incised product (P). Data are expressed as the means ± SD of three technical replicates from two independent assays. A representative image of a denaturing polyacrylamide gel analysis of the enzymatic reactions is shown. NE, no cell extract; NT, non-treated cells. Asterisks represent a significant difference with respect to control (NT).* P<0.05, ** P< 0.001.

b) APE1 protein level evaluated in HeLa cells treated with 20 µM compound #3, 40 µM fiduxosin (FDX) or 100 µM E3330 for 24 h, and in HeLa cells silenced for APE1 expression (siRNA). Representative Western blotting analyses of total cell extracts of HeLa cell probed with APE1 antibody are shown. Tubulin was used as loading control and for data normalization. NT, non-treated cells.

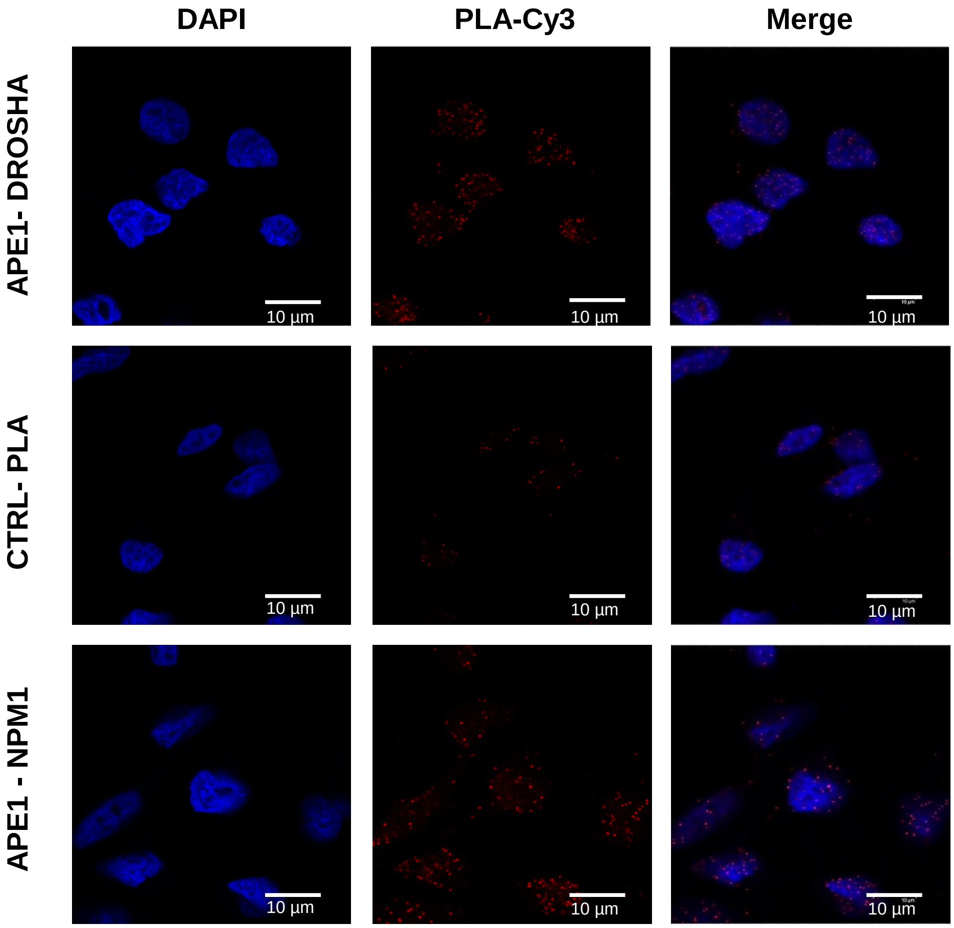

Supplementary Figure 4

a) Nucleoplasmic interaction between APE1 and DROSHA complex. PLA technology[23] was used to evaluate *in vivo* the APE1-DROSHA interaction. PLA reaction was performed following manufacturer's instructions. HeLa cells were seeded on a glass coverslip and PLA reaction was carried out using anti-APE1 and anti-DROSHA antibodies. Confocal microscopy analysis highlighted the presence of distinct fluorescent red dots (PLA signals) indicating the occurrence of *in vivo* interaction between APE1 and DROSHA. DAPI staining was used as a reference for the nuclei. Negative control is represented by cells incubated only with α-APE1 antibody (Ctrl-PLA). Positive control is represented by APE1 and NPM1 interaction as already established[1]. Bars, 10 μM.

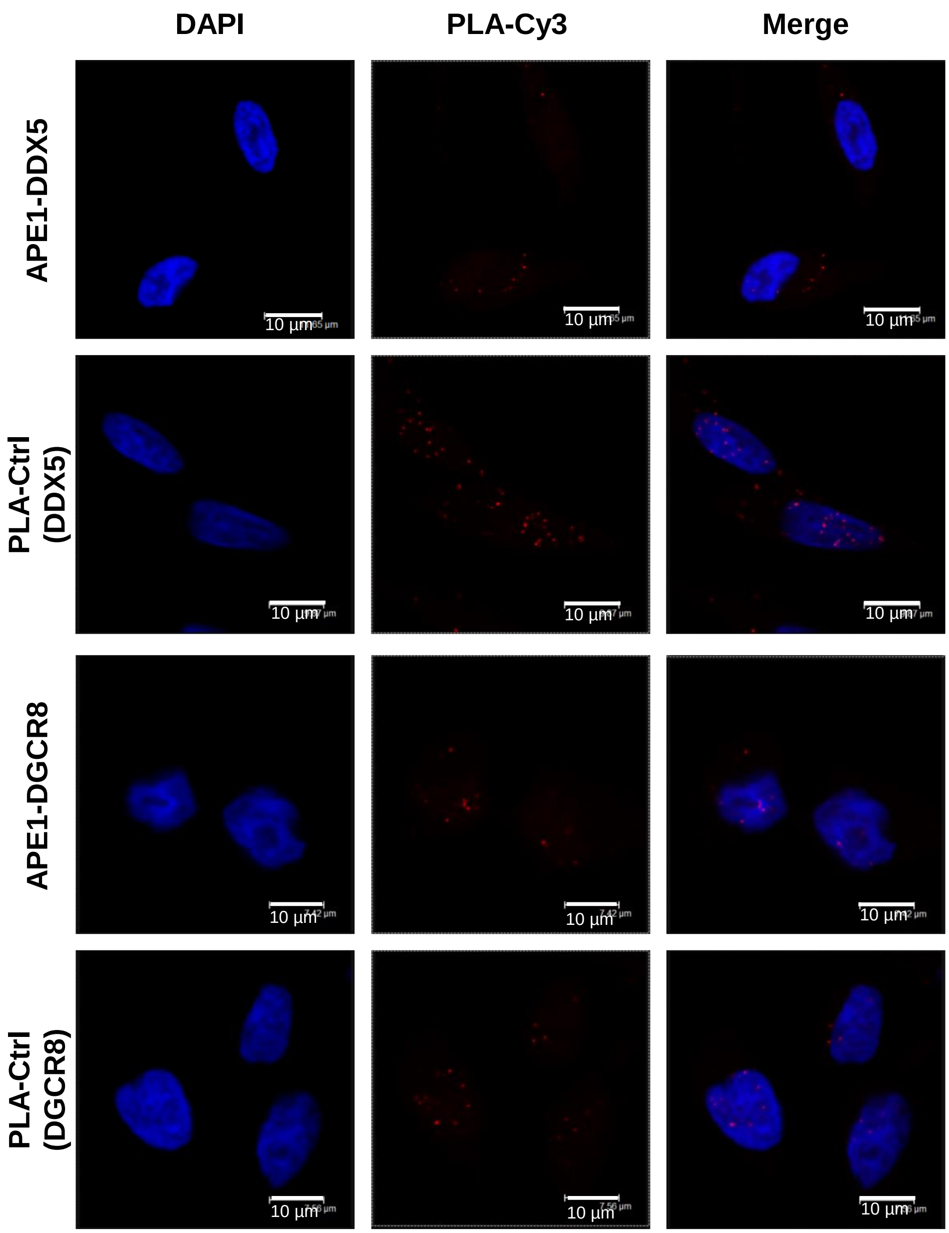

Supplementary Figure 4

b) Nucleoplasmic interaction between APE1, DDX5 and DGCR8. PLA technology was used to evaluate *in vivo* APE1-DDX5 and APE1-DGCR8 interaction. PLA reaction was performed following manufacturer's instructions. HeLa cells were seeded on a glass coverslip and PLA reaction was carried out using anti-APE1 and anti-DDX5 (ab10261) or anti-DGCR8 (ab191875) antibodies. DAPI staining was used as a reference for the nuclei. Negative control is represented by cells incubated only with α-DDX5 or α-DGCR8 antibody (Ctrl-PLA). Bars, 10 µM.

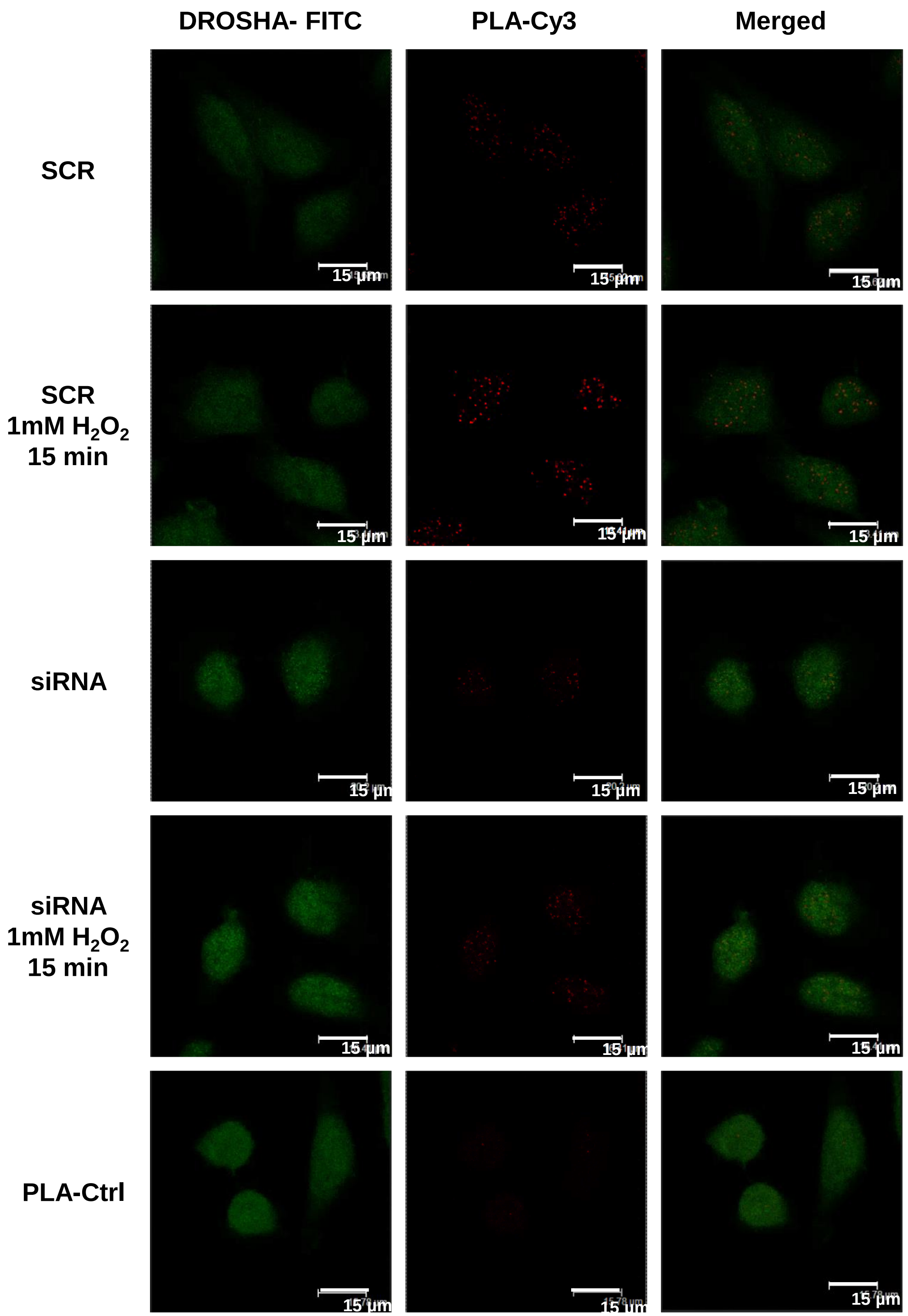

Supplementary Figure 4

c) Nucleoplasmic interaction between APE1 and the DROSHA complex after oxidative stress in HeLa cell clones silenced for APE1 expression. HeLa cell clones stably transfected with scrambled siRNA control (SCR) or with an APE1 siRNA (siRNA) were seeded on a glass coverslip and treated with 1 mM H_2O_2 for 15 min. PLA reaction was carried out using anti-APE1 and anti-DROSHA antibodies. DROSHA expression was detected by using an anti-DROSHA antibody and was used as a reference for the nuclei. Negative control is represented by cells incubated only with α-DROSHA antibody (Ctrl-PLA). Bars, 15 µM.

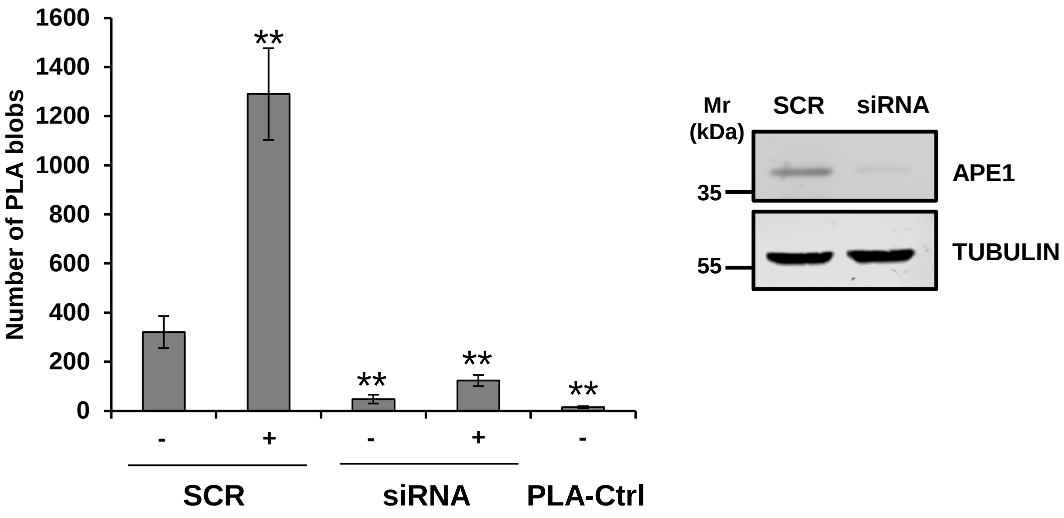

Supplementary Figure 4
d) Data reported in the histogram account for the average number of PLA signals of at least 30 randomly selected cells per condition. ** P<0.001. Right, representative Western blotting analyses to confirm APE1 silencing in HeLa cell clone extracts silenced for APE1 expression.

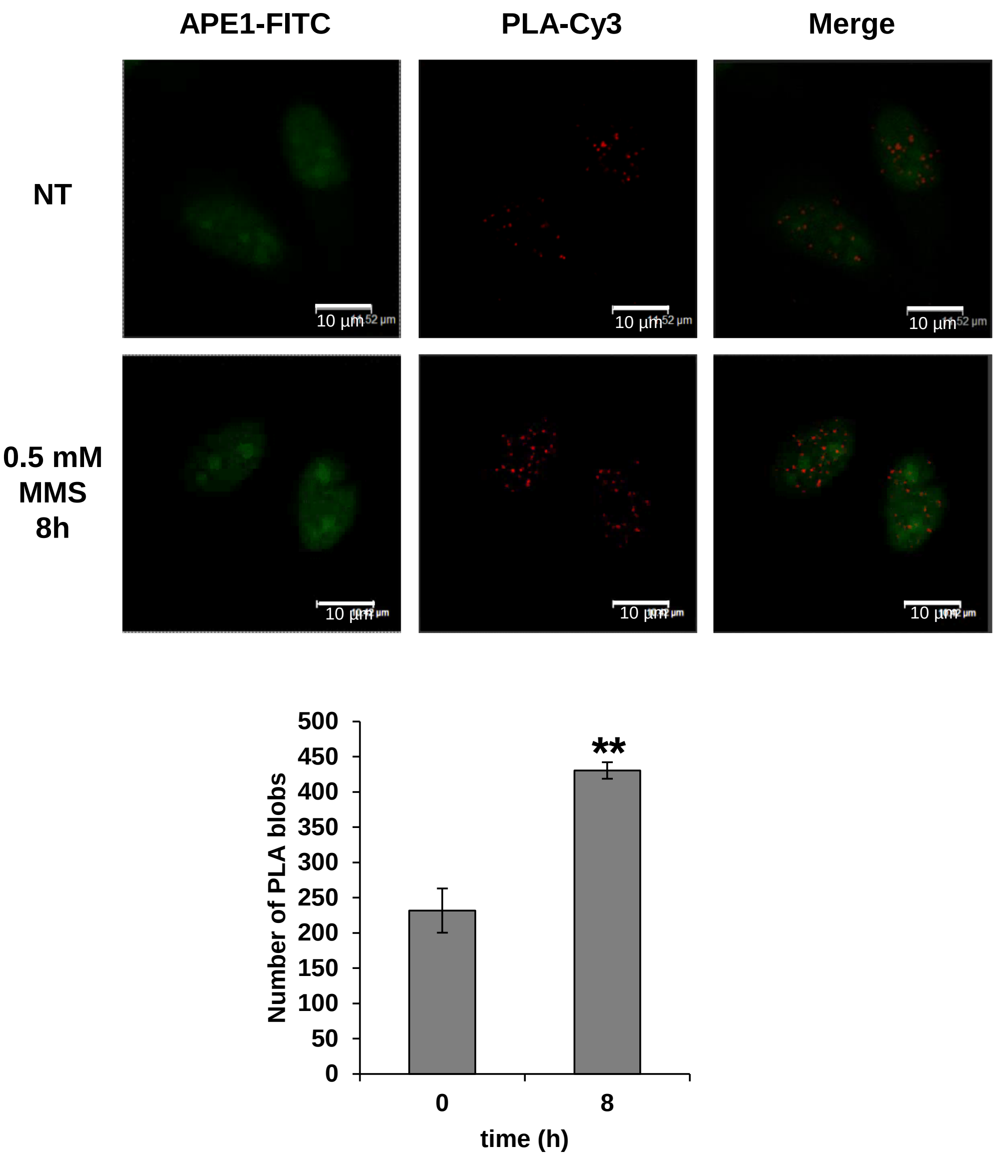

Supplementary Figure 4

e) Nucleoplasmic interaction between APE1 and DROSHA complex after genotoxic stress. HeLa cells were seeded on a glass coverslip and treated with 0.5 mM methyl methanesulfonate (MMS) for 8 h. PLA reaction was carried out using anti-APE1 and anti-DROSHA antibodies. APE1 protein expression was detected by using an anti-APE1 antibody and was used as a reference for the nuclei. Data reported in the histogram accounted for the average number of PLA signals of at least 30 randomly selected cells per condition. ** $P<0.001$.

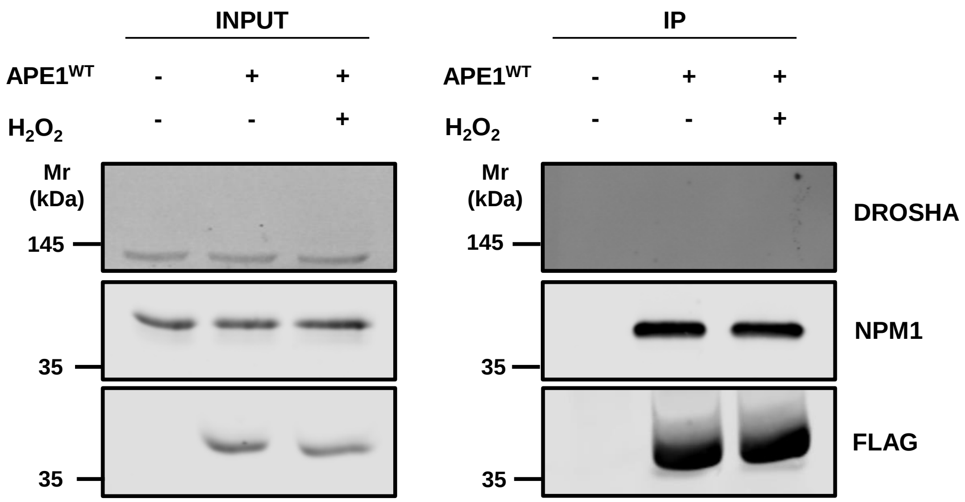

Supplementary Figure 4

f) Coimmunoprecipitation (CoIP) analysis on HeLa cells transfected with APE1 WT FLAG-tagged proteins with endogenous DROSHA after treatment with 1mM H_2O_2 for 15 min. Total cell extracts were immunoprecipitated with FLAG antibody and Western blot analysis was used to quantify the interaction among APE1 and DROSHA. Western blot analysis was performed on total cell extracts (left) and on immunoprecipitated material (right) with specific antibody for endogenous DROSHA and FLAG for APE1 transfected.

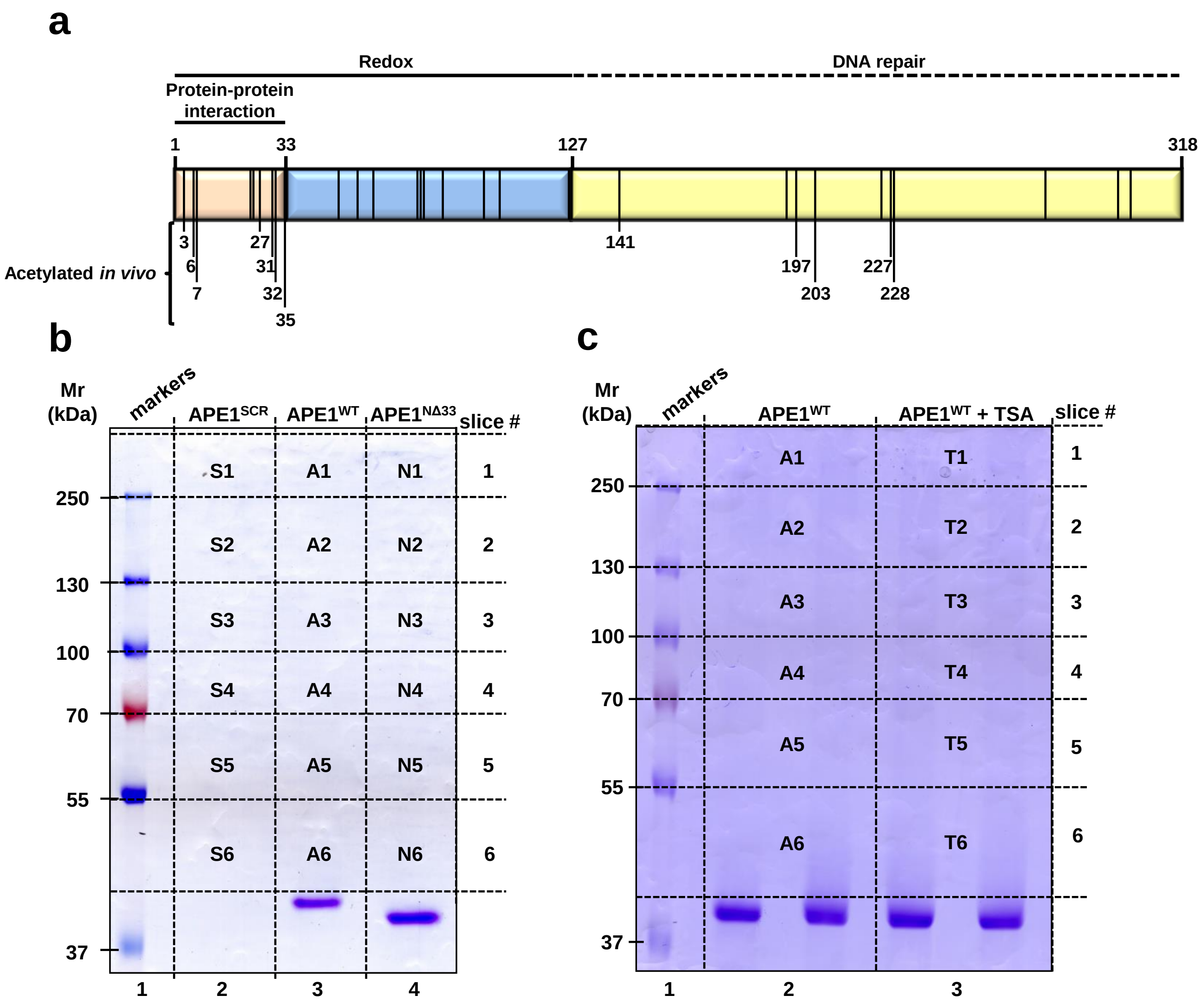

Supplementary Figure 5

a) Domain structure of APE1 protein and identification of APE1 acetylation sites *in vivo*. The first 33 residues (orange), which are important for nuclear localization, RNA binding and protein-protein interaction, occur within the N-terminal portion of the protein (blue), also devoted to redox regulation of different transcription factors. The C-terminal portion (yellow) contains the catalytic residues, essential for the DNA repair activity within the BER pathway. Within the protein structure, all the lysine residues are highlighted (vertical black lines); the acetylated lysine residues identified by MS are numbered.

b) and **c)** Coomassie-blue staining of SDS-PAGE resolved immunoprecipitated material for MS identification of APE1 interacting partners. SDS-PAGE gel of co-immunopurified complexes from cell clones, expressing flag tagged APE1^{WT} and N-terminus deletion mutant (APE1$^{N\Delta33}$) (b) and APE1 WT cells after trichostatin A (TSA, 0.3 mM for 4 h) treatment (c). Vertical and horizontal axes indicate apparent molecular mass (kDa) and clone/treatment considered for each lane, respectively. Control represents HeLa cells stably transfected with a scrambled shRNA sequence (APE1^{SCR}), APE1^{WT} represents cells expressing only the ectopic APE1 protein in spite of the endogenous one, while APE1^{ND33} cells expressing the ectopic APE1 deletion mutant.

d

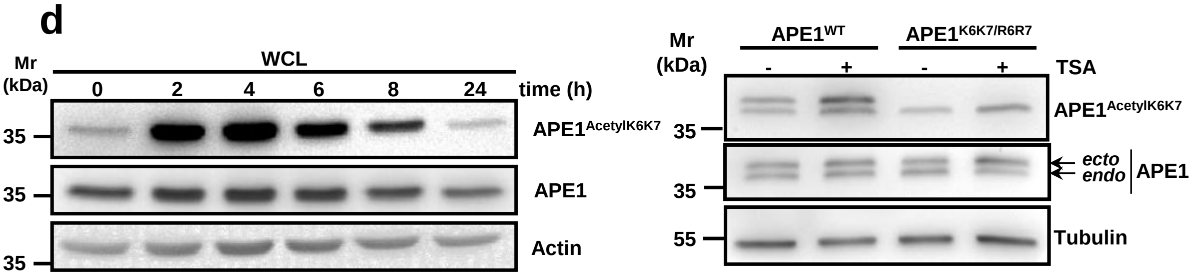

e

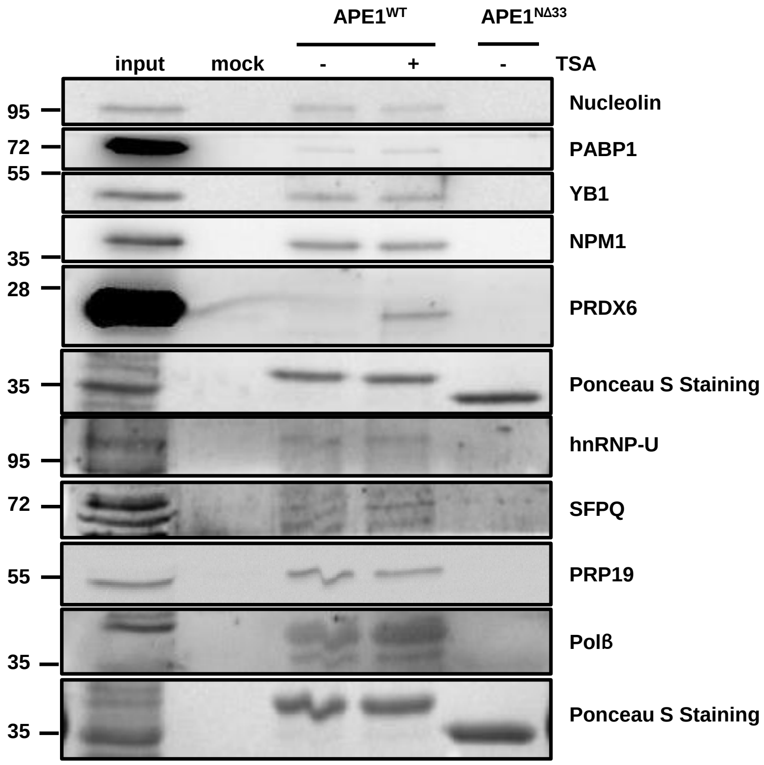

Supplementary Figure 5

d) TSA treatment increases APE1 acetylation at K6/K7. Representative Western blotting analysis on HeLa cells treated with 0.3 mM TSA for the indicated times (left) and on cell clones expressing $APE1^{WT}$ and a non-acetylatable mutant of the protein in K6/K7 ($APE1^{K6K7/R6R7}$) treated with 0.3 mM TSA for 4 h (right). Data show the increased acetylation at APE1 K6/K7 following TSA treatment. Levels of endogenous (endo) or ectopic (ecto) proteins were detected with specific antibodies, as indicated on the right-hand side. Either actin or tubulin was used as loading control. Developed by using the ECL enhanced chemiluminescence procedure.

e) Validation of new APE1 interactors identified by mass spectrometry. Co-immunopurified material from $APE1^{WT}$ and $APE1^{N\Delta33}$ cell clones treated with TSA (0.3 mM, 4 h) was separated onto SDS-PAGE and Western blotting analysis was performed to validate the APE1-interacting partners identified through MS. Ponceau S staining was used as loading control. The filters were developed by using the ECL enhanced chemiluminescence procedure.

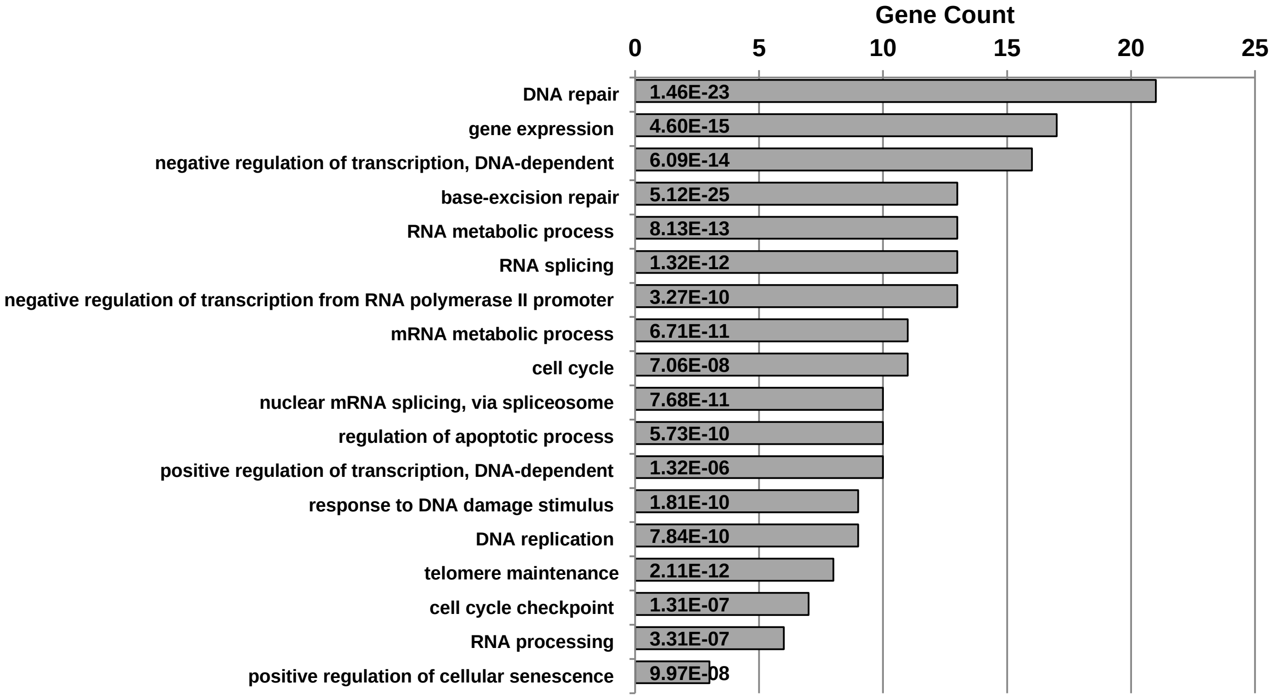

Supplementary Figure 6

a) Functional enrichment analysis of APE1-interacting protein species. GeneCodis analyses of all (103) known APE1-interacting partners as retrieved in literature. Data show enrichment according to molecular function. For the sake of clarity, only the most representative functional categories are shown. The number of genes for each category is provided on horizontal axis and list only the first five co-occurrence terms. Statistical significance belonging to each category is reported within each bar.

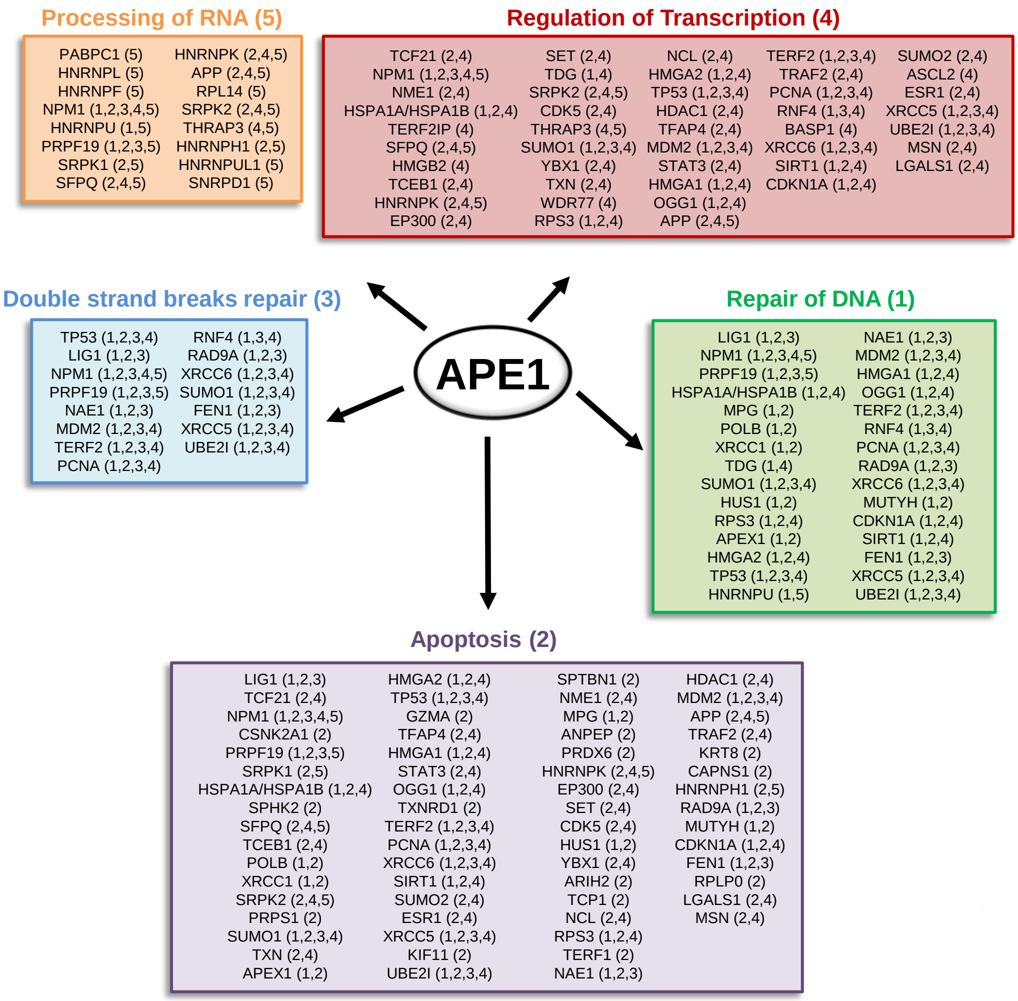

Supplementary Figure 6

b) Representation of top five functional annotation clusters of APE1-interacting proteins identified by Ingenuity Pathway Analysis based on functional terms of the “biological process” category. Protein targets occurring in more than one cluster are cross-referenced with numbers.

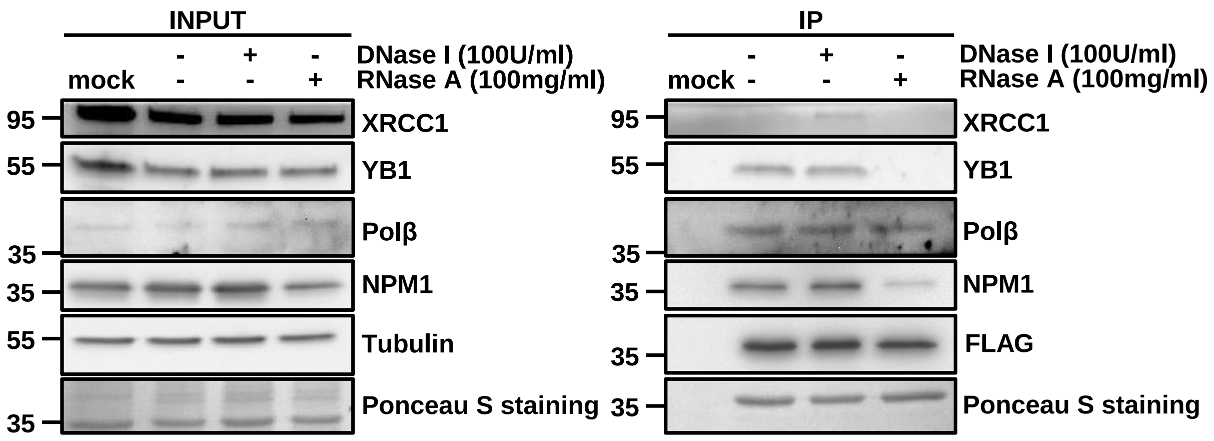

Supplementary Figure 7
Representation of top five functional annotation clusters of APE1-interacting proteins identified by Ingenuity Pathway Analysis based on functional terms of the "biological process" category. Protein targets occurring in more than one cluster are cross-referenced with numbers.

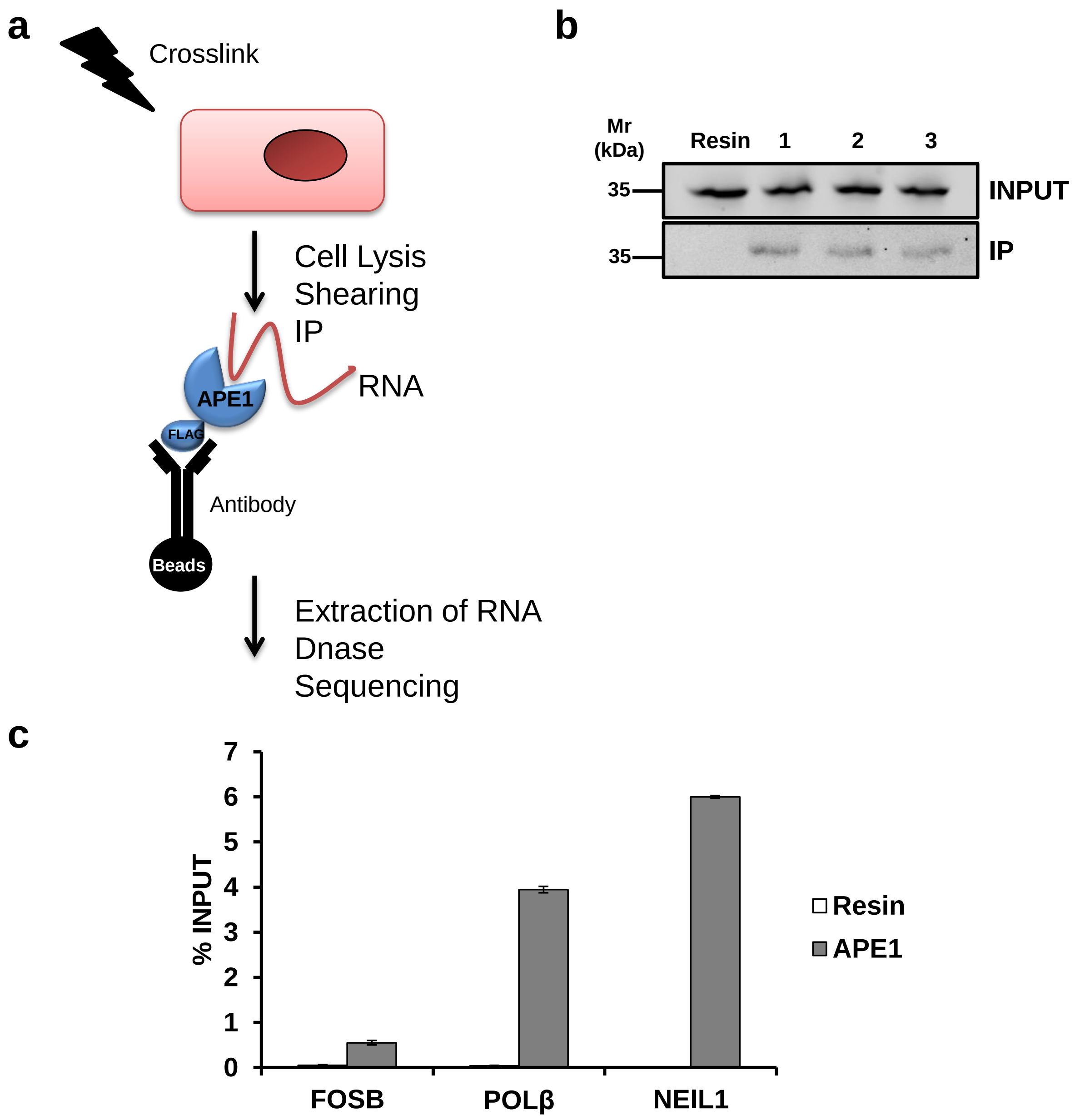

Supplementary Figure 8

a) Schematic representation of APE1 RIP with HeLa cell clones expressing the APE1^{WT} protein fused to a FLAG tag. Protein-RNA complexes were formaldehyde-crosslinked and cells were lysed; RNA was trimmed by sonication and complexes were immunoprecipitated using antibody against FLAG tag or control resin without the antibody (see Supplementary Methods for further details).

b) Representative Western blotting to confirm APE1 pulldown in the RIP experiment. Western blotting analysis was performed on total HeLa cell clone extracts (input) and on immunoprecipitated material (IP) with specific antibody for FLAG. Resin, HeLa cell clone extracts immunoprecipitated with resin lacking the anti-FLAG antibody; 1-3, three different replicates.

c) Real-time PCR validation of three RNA targets identified through RNA-sequencing analysis. Data are presented as fold percentage of the amount of immunoprecipitated target RNA relative to that present in total input RNA. Resin, HeLa cell extracts immunoprecipitated with resin lacking the anti-FLAG antibody; APE1, immunoprecipitated material derived from a pool of three replicates.

RIP-seq DATA Functional analysis

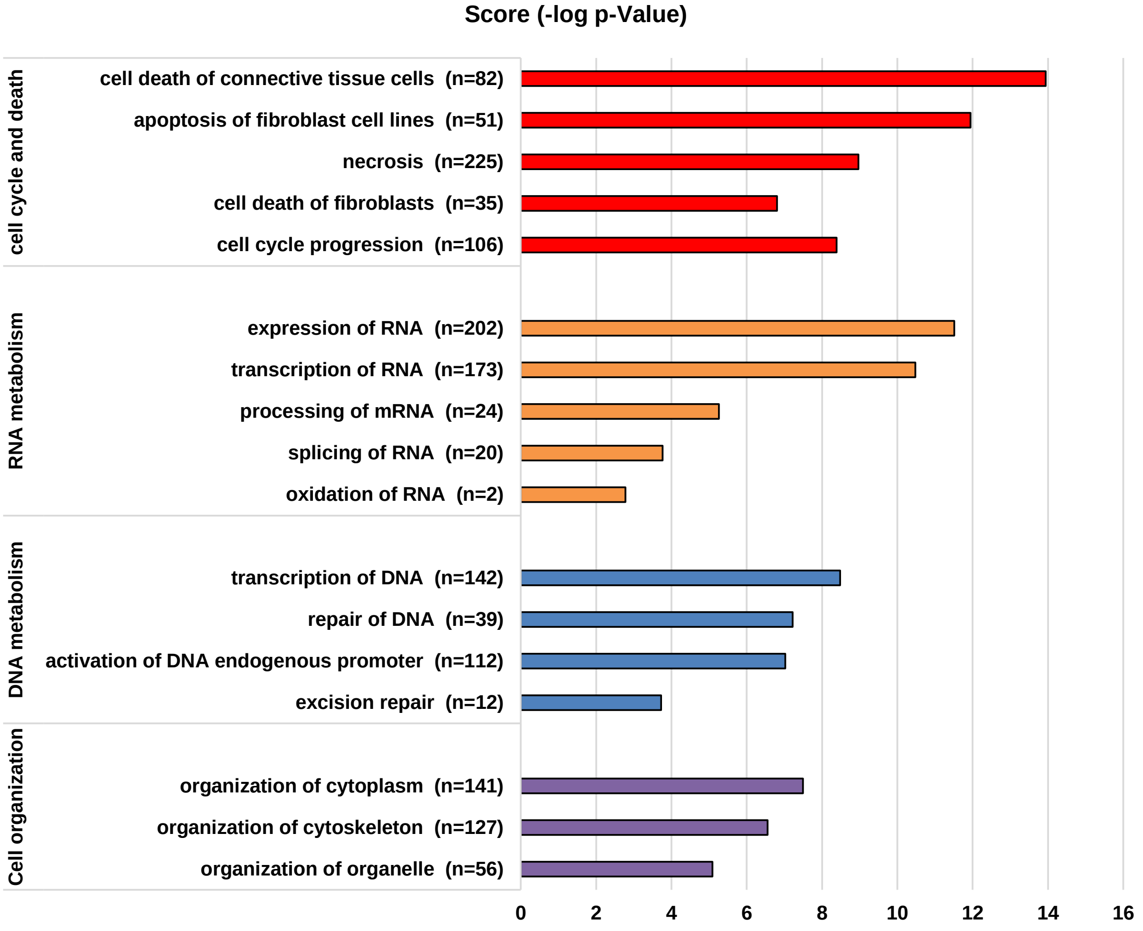

Supplementary Figure 8

d) Bar chart showing the enrichment of specific functions obtained using IPA for the APE1-RNA interactome. Bars length represents the –(log p-value) of the enrichment. The number of genes down-regulated in the experiment and annotated for every specific function is shown.

a

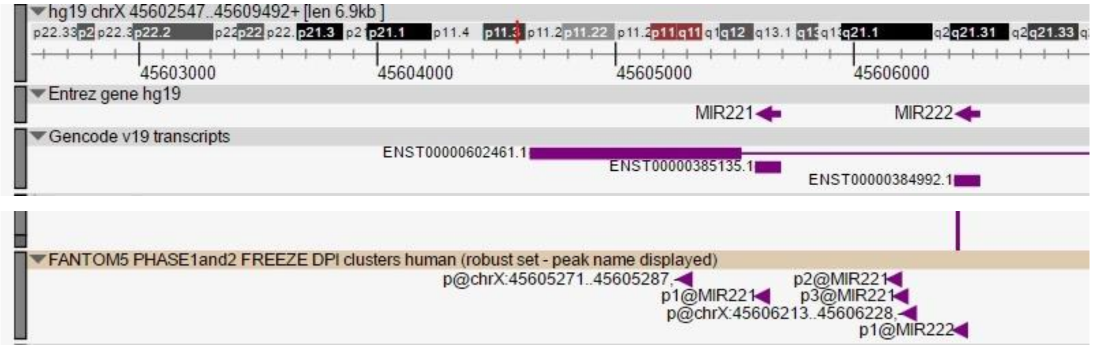

b

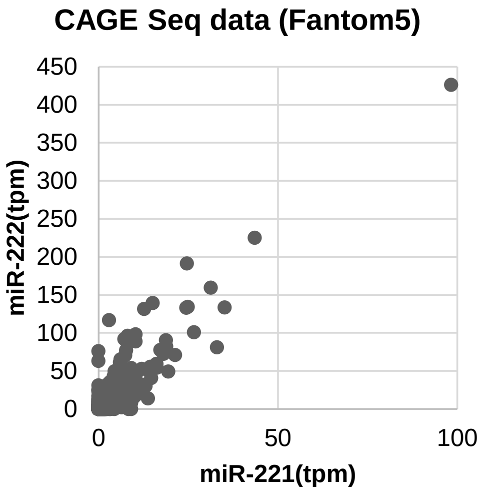

c

	miR-221 (tpm)	miR-222 (tpm)
**HelaS3 rep1 (CNhs12325)**	0.774	1.316
**HelaS3 rep3 (CNhs12327)**	0.411	1.258
**HelaS3 rep2 (CNhs12326)**	0.146	1.068

Supplementary Figure 9

a) Modified screenshot of the genomic region for miR-221 and miR-222 in ZENBU Genome Browser (http://fantom.gsc.riken.jp/zenbu/gLyphs/) showing the presence of independent different CAGE-seq peaks for the two miRNAs).

b) Scatter plot of CAGE-seq peak activities for miR-221 and miR-222 in the FANTOM5 sample collection (n=1830).

c) Table showing the CAGE-seq peak activities for miR-221 and miR-222 in the HeLa cells.

Fig. 2b

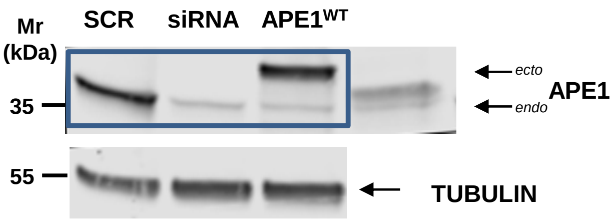

Fig. 2c

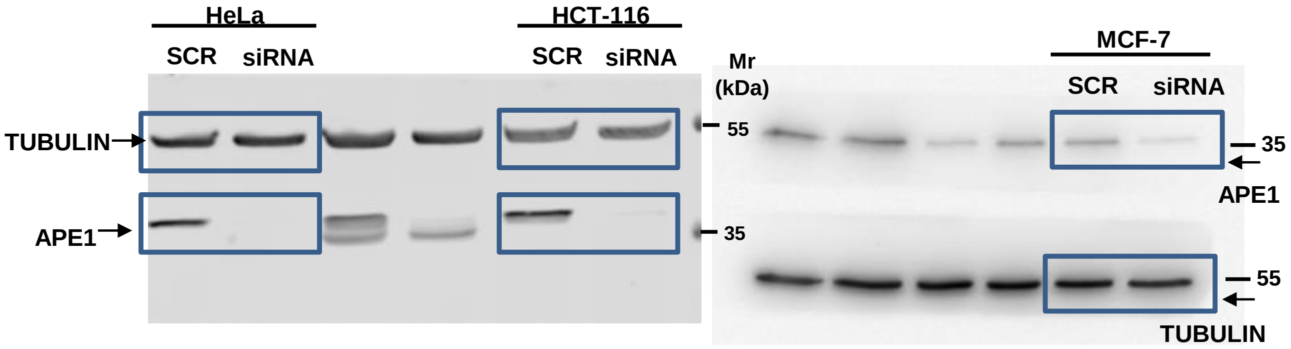

Fig. 3b

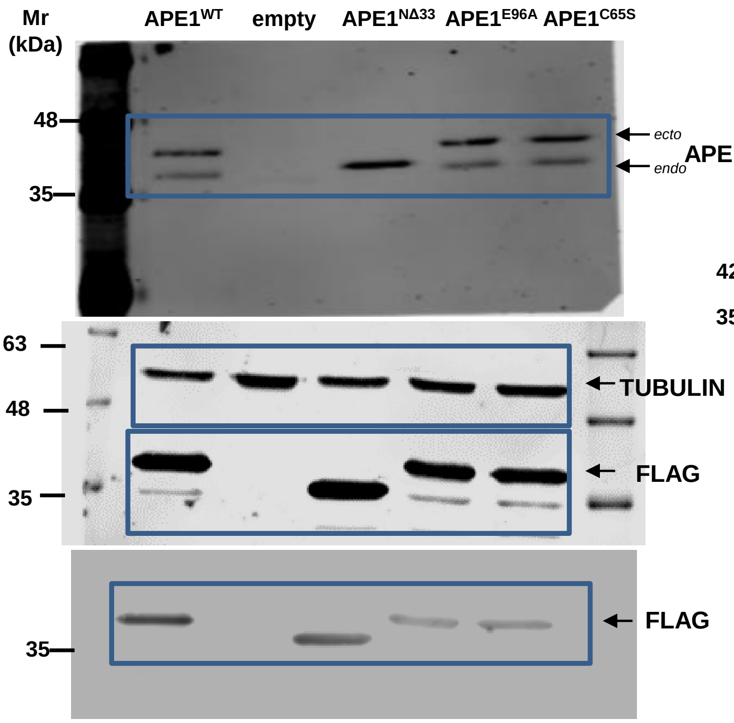

Fig. 3d

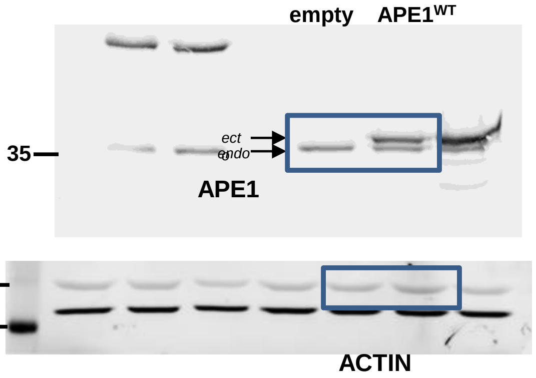

Supplementary Figure 10
Uncropped blots for Figure 2 and Figure 3

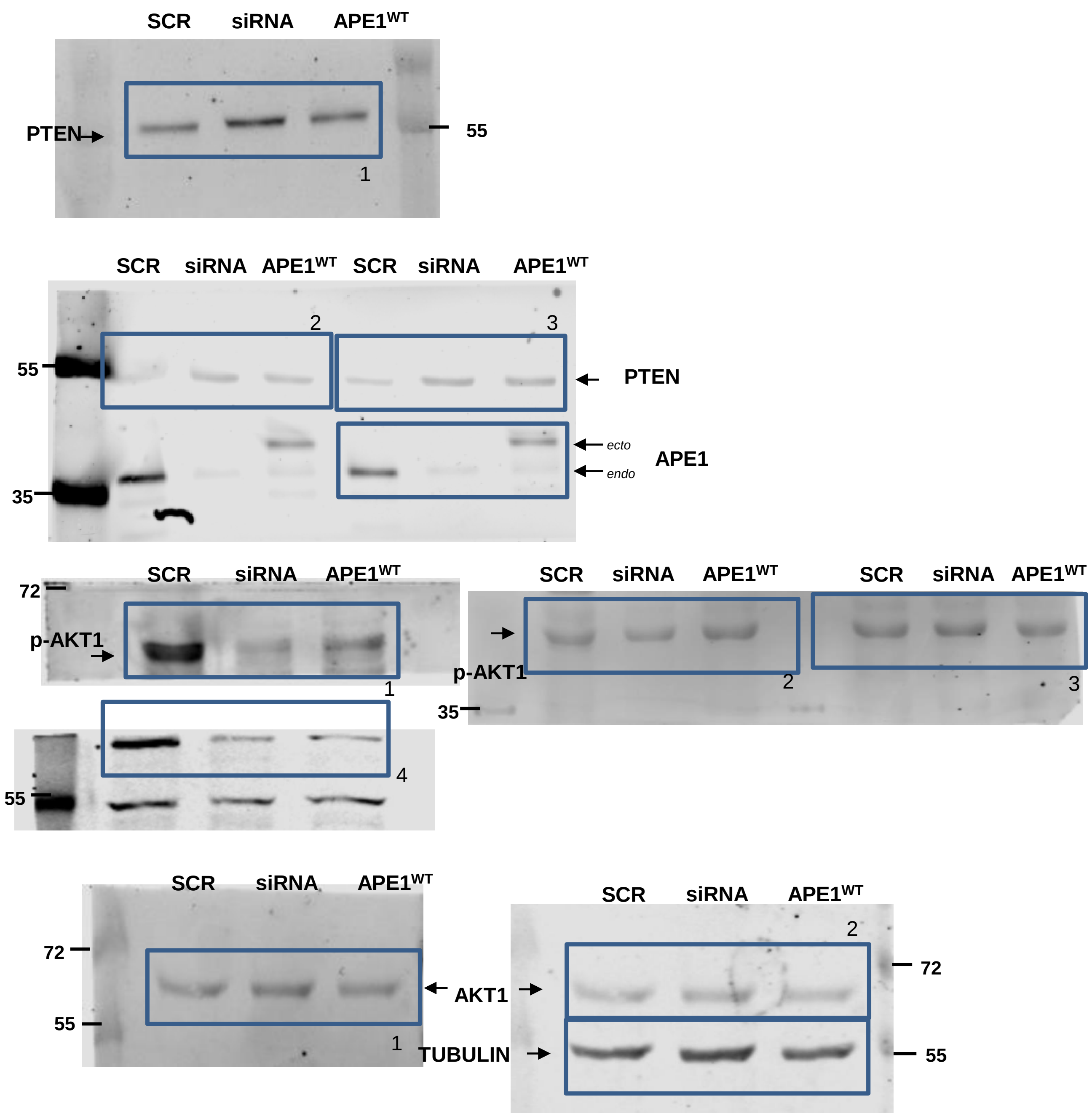

Supplementary Figure 11
Uncropped blots for Figure 5c

Supplementary Tables

Supplementary Table 1. The most significant differentially expressed miRNAs respectively in H_2O_2 vs SCR and siAPE1 vs SCR comparisons. Fold-change in logarithmic scale, significance (pValue) and corrected pValue (qValue) are shown.

SCR H_2O_2 vs SCR			
**ID**	**logFC**	**p-value**	**q-value**
**hsa-miR-221-3p**	1.36	8.27E-14	3.31E-11
**hsa-miR-20a-5p/20b-5p**	1.00	4.57E-14	3.31E-11
**hsa-miR-1246**	-1.48	2.60E-12	6.33E-10
**hsa-let-7f-5p**	1.09	4.63E-11	7.41E-09
**hsa-miR-16-5p**	1.06	8.86E-11	1.18E-08
**hsa-miR-30c-5p**	1.49	5.94E-10	6.79E-08
**hsa-miR-200c-3p**	1.79	1.03E-08	7.46E-07
**hsa-miR-378e**	1.59	1.89E-08	1.16E-06
**hsa-miR-26a-5p**	1.43	1.87E-08	1.16E-06
**hsa-miR-30b-5p**	1.08	2.67E-08	1.53E-06

siRNA APE1 vs SCR			
**ID**	**logFC**	**p-value**	**q-value**
**hsa-miR-301a-3p**	-1.80	3.89E-12	3.11E-09
**hsa-miR-23b-3p**	-1.60	1.21E-10	2.86E-08
**hsa-miR-107**	-1.75	1.18E-10	2.86E-08
**hsa-miR-505-3p**	-2.45	1.43E-10	2.86E-08
**hsa-miR-148b-3p**	-1.37	8.60E-10	1.38E-07
**hsa-miR-23a-3p**	-1.38	2.05E-09	2.74E-07
**hsa-miR-99b-5p**	-1.30	6.44E-09	7.36E-07
**hsa-miR-4286**	-6.25	8.90E-09	8.90E-07
**hsa-miR-324-5p**	-1.44	1.88E-08	1.67E-06
**hsa-miR-365a-3p**	-1.51	4.23E-08	3.39E-06

Supplementary Notes

Identification of the *in vivo* APE1 acetylation sites

Previous studies from this and other laboratories have already demonstrated that protein acetylation occurs at K3, K6, K7, K27, K31, K32, K35 and K197[3,7,8]. Since these investigations were elusive in detecting concomitant modification at these residues, we decided to deeply investigate protein acetylation by integrating our previous peptide mapping analysis performed using endoprotease AspN[1,2] with data obtained after digesting APE1 with proteases having different substrate specificity (trypsin and endoprotease LysC). Best results were obtained in the case of trypsin (Supplementary Data File 4), which confirmed modification at K27, K31, K32, K35 and K197, and originally proved acetylation at K141, K203, K227 and K228 (Supplementary Data File 3 and Supplementary Fig. 4a). No additional information derived from the experiments performed with endoprotease LysC (data not shown). A visual inspection of the crystallographic structures of APE1 present in PDB demonstrated that all these residues are present on the molecular surface (data not shown), highly accessible to protein acetylase and deacetylase activities. Notably, a significant percentage (58%) of the acetylation sites fall within the 35 amino acids present at the protein N-terminus, remarking the importance of this region in regulating APE1 functions[1,2]. Indeed, acetylation, which occur on residues close to the protein cleavage site (K31), may modulate its susceptibility to proteolytic degradation and may affect APE1 binding to its interacting partners[1].

Characterization of the APE1 protein-interactome and role of APE1 acetylation in controlling APE1 equilibrium with its protein interacting partners

We previously characterized the APE1 interactome under basal conditions through a proteomic approach based on the resolution of APE1-immunopurified complexes through two-dimensional electrophoresis[9]. This approach had the limitation of a poor output for high molecular mass and basic proteins. In order to extend our interactomic analysis, immunopurified complexes from HeLa cells reconstituted with a Flag-tagged APE1 form ($APE1^{WT}$)[4,8] were directly resolved through SDS-PAGE; analysis was performed focusing on proteins migrating in the molecular mass range 50-300 kDa (Supplementary Fig. 4b,c). As a control, we used immunopurified material from HeLa cells stably transfected with the empty vector and expressing a scrambled siRNA sequence ($APE1^{SCR}$)[8]. Whole gel lanes were cut into contiguous gel portions that were directly subjected to proteomic analysis; lists of identified proteins from $APE1^{WT}$ were subtracted of components detected in control counterparts (Supplementary Data File 5). This approach has been successfully used in our laboratory for other protein interactomic studies[10–12]. Besides confirming several APE1-binding proteins previously characterized, which are highlighted in italics in Supplementary Data File 2[9], nineteen novel interacting partners were identified. Among the latter protein species, nine are involved in RNA metabolism (RL14, RL3, RL4, hnRNP-F, hnRNP-H, hnRNP-U, NCL, PABP1 and YB1), five in cytoskeleton dynamics (ACTN-1, KIF11, MOES, MYH9 and MYO1C), one in secretion (SPTB2) and one in proteolysis (APN). Interestingly, two (BASP1 and THRAP3) are involved in transcriptional regulatory processes and one (SFPQ) plays a role both in RNA metabolism/transcription activation and in NHEJ repair of DNA double strand breaks[13,14]. Validation experiments were performed by Western blotting analyses on an independent set of immunoprecipitated material (Supplementary Fig. 4e and data not shown).

In order to evaluate the effect of the protein N-terminal region on APE1 molecular interactions, a comparative interactomic analysis was performed with HeLa cells reconstituted with a Flag-tagged

protein form lacking the initial 33 amino acids present at the N-terminus (APE1$^{N\Delta33}$) (Supplementary Fig. 4b,e). As already demonstrated for other proteins[9], particularly evident was the loss of binding of APE1$^{N\Delta33}$ to seven novel binding partners (RL4, KIF11, MOES, NCL, SFPQ, THRAP3 and YB1) (Supplementary Data File 2 and 5). Only in the case of BASP1, the N-terminal sequence was dispensable for the interaction with APE1.

Previous data from our laboratory demonstrated a negative impact of acetylation at K27-K35 on APE1 interaction with rRNA and NPM1[8]. To determine the possible role of acetylation in mediating APE1 protein-protein interaction, the effect of TSA, a HDAC inhibitor known to induce APE1 hyperacetylation at K6 and K7[15,16], was then assayed with respect to the nature of the APE1 interactome (Supplementary Fig. 4c,e). The time of TSA treatment was chosen on the basis of kinetics experiments (Supplementary Fig. 4d, left) in which we quantitatively evaluated the extent of K6/K7 acetylation by Western blot analysis, using a specific antibody raised toward this APE1 acetylated form (Supplementary Fig. 4d, right)[15,16]. Upon 4 h of TSA treatment, the acetylation level of K6/K7 was maximal (Supplementary Fig. 4d, left). Regarding APE1 association, two groups with opposite behavior were observed: six proteins (RL14, hnRNP-H, hnRNP-U, MYH9, MYO1C and SPTB2) showed an increased interaction upon TSA treatment, while three (RL3, RL4 and PABP1) presented an opposite trend (Supplementary Data File 2 and 5). In addition, validation experiments on the effect of the truncation at the protein N-terminus and of the TSA treatment were performed by Western blotting analyses on an independent set of immunoprecipitated material (Supplementary Fig. 4e and data not shown).

Taken together with the data obtained in our previous work[9], these results allowed compiling a list of APE1-interacting partners (28 in number) (Supplementary Data File 2) that, surprisingly, does not

contain classical DNA-repair enzymes involved in BER (such as Polβ or XRCC-1), which are well known to interact with APE1[17,18]. This may be essentially due to the low representation of these DNA repair enzymes in mammalian cells (with respect to other APE1-binding proteins), and the sensitivity of the method we used, able to recognize only the most abundant components. For the majority of protein-interacting partners reported in Supplementary Data File 2, the interaction seemed to require the N-terminal domain of APE1 and is affected by the TSA treatment, further emphasizing that acetylation may modulate an effective mutual binding.

With the aim of expanding our analysis to all known APE1 interacting partners (Supplementary Data File 6), an enrichment of GO terms related to protein and nucleic acids binding, but also to DNA repair and gene expression, was observed (Fig. 7 and Supplementary Fig. 5). All these data reinforce the overall idea that APE1 may act as a multifunctional hub protein, emphasizing the emerging role of APE1 in RNA metabolism.

APE1 interactome is mediated by RNA molecules

Several evidence support the concept that nucleic acids, in particular RNA molecules, are important mediators of the interaction between DNA repair proteins and their interacting partners[8,19,20]. Therefore, as paradigmatical examples, we checked whether the APE1 interactions with a protein involved in RNA binding (YB-1) and in DNA repair (Polβ and XRCC1) require RNA and/or DNA molecules. APE1 association with its interacting partners was not mediated by DNA since it could be detected in DNase I-treated immunoprecipitated samples (Supplementary Fig. 5a). By contrast, treatment with DNase-free chromatographically purified RNase A mostly reduced it. These data

suggest that the strong interaction occurring between APE1 and some of its interacting partners may involve RNA molecules, as previously described for the APE1-NPM1 interaction[20].

Bioinformatics evidence of independent transcription of miR-221 and miR-222

It is commonly thought that miR-221/222 belong to a polycistronic cluster giving rise to a unique pri-miRNA sequence. However, the transcriptional regulation of this cluster has not been completely elucidated, yet. In fact, data from both experimental and ENCODE analyses[21] revealed that this cluster may be originated from different transcripts of different lengths (i.e. 5.6, 28.2 and 108.5 kb), which contain the sequences for mature miR-221/222 around 3-4 kb; no specific characterization of the promoter regions has been published yet. Our data, showing different expression levels for the two pri-miRNAs, suggested that an additional Transcription Start Site (TSS) may be present in the spacer region between the two pri-miRNA sequences.

In order to try to understand if multiple TSS were indeed present in the miR-221/222 locus, we used the atlas of promoter activities released by the FANTOM5 project[22] to measure TSS and the promoter usage across a collection of over 1800 human samples. This approach allowed to define two different regions of transcription initiation, one associated with the expression of miR-221 (hg19::chrX:45605575-45606248) and a second one associated with miR-222 (hg19::chrX:45606341-45606513); see Supplementary Fig. 9a.

In the 1174 human samples where both miRNAs are expressed, the RLE-normalized promoter activity of miR-222 is on average 3.8-fold higher than that of miR-221 (median value = 2.8-fold)

(Supplementary Fig. 9b). Moreover, there are 284 samples where only miR-222 is expressed and 74 samples where only miR-221 is expressed, and 297 samples where both samples are not expressed. We also restricted this comparison to the HeLa cell line, as we used in this manuscript, confirming the higher promoter activity of miR-222 (average RLE-normalized expression of pri-miR222 4.4-fold higher than pri-miR221, median RLE-normalized expression of pri-miR222 2.6-fold higher) (Supplementary Fig. 9c). All these data perfectly matched with our observations, which show higher expression level of pri-miR-222 than pri-miR-221 (Average expression of pri-miR-222 3.5-fold ± 0.12 higher than pri-miR-221) in the HeLa cell line we used.

Therefore, it is possible that the difference we observed in the pri-miRNA expression levels under basal conditions (Fig. 2c) and under oxidative stress conditions (Fig. 4b) could be ascribed to the use of alternative promoters, leading to the expression of different levels of the two pri-miRNAs molecules. Further work is required to address this interesting issue.

Supplementary Methods

Co-immunoprecipitation

Co-immunoprecipitation studies were carried out with whole cell extracts from HeLa cell. 24 h upon transfection with FLAG-tagged APE1 wild-type protein encoding plasmid, cells were treated with 1mM H_2O_2 for 15 min and harvested. Cells were then washed twice with PBS and resuspended in lysis buffer (50 mMTris–HCl pH7.4, 150 mM NaCl, 1 mM EDTA and 1% Triton X-100)containing proteases inhibitor cocktail. After incubation for 20 minutes at 4°C under rotation, cell lysates were clarified by centrifugation at 12,000 × g for 10 minutes at 4°C and coimmunoprecipitation was performed with anti-FLAG M2 affinity gel (SIGMA-ALDRICH, Milan, Italy) at 4°C with gentle rocking for 3 hours. After washing three times with Tris-buffered saline (TBS), immunoprecipitates were then eluted by incubation with 0.15 mg ml^{-1} FLAG peptide in TBS and analyzed as indicated.

Samples were then loaded onto a 12 w/vol % SDS-PAGE electrophoresis gel. Proteins were then transferred to nitrocellulose membranes (Schleicher & Schuell, Keene, NH, USA). Monoclonal α-Ape1 was from Novus Biologicals (Littleton, CO, USA-NB 100-116), polyclonal anti-DROSHA was from Abcam (ab85027, Abcam, Cambridge, MA) and anti-FLAG from SIGMA (F1804). Monoclonal α-NPM1 from Invitrogen (Carlsbad, California, USA - 32-5200) was used as IP positive control. Membranes were incubated with secondary antibodies labeled with IRDye (1:10,000 dilution) in 5% milk, PBS and Tween 0.1%. All gel images were captured with an Odissey CLx scanner (LI-COR GmbH, Germany) and analyzed using the ImageStudio software (LI-COR GmbH, Germany).

Antibodies for Western Blotting

Monoclonal α-APE1 was from Novus (NB 100-116) (1:1000); monoclonal α-NPM1 was from Invitrogen (32-5200) (1:1000); monoclonal α-XRCC1 was from Thermo Scientific (MS-434-P0) (1:1000); polyclonal α-YB1 was from Abcam (ab12148) (1:1000); polyclonal α-Polβ was from Abcam (ab26343) (1:1000); monoclonal α-FLAG was from Sigma (F1804) (1:1000); polyclonal α-PRP19 was from Abcam (ab27692) (1:1000); monoclonal α-Nucleolin was from Zymed (39-6400) (1:1000); polyclonal α-SFPQ was from Abcam (ab38148) (1:1000); monoclonal α-PABP-1 was from abcam (ab6125) (1:1000); monoclonal α-PRDX6 was from Abcam (ab16947) (1:1000); monoclonal α-hnRNP-U was from Abcam (ab89413) (1:1000). Polyclonal α-Actin and monoclonal α-Tubulin were from Sigma (A2066 and T-9026, respectively) (1:2000). Polyclonal α-APE1 Acetyl K6K7 was a kind gift from Prof. Kishor K. Bhakat (University of Nebraska Medical Center, Omaha, USA) (1:1000).

Protein acetylation analysis

Identification of the acetylated K residues in APE1 was performed on immunopurified APE1 WT protein ($APE1^{WT}$), which was subjected to SDS-PAGE, excised, S-alkylated with iodoacetamide and digested *in-gel* with trypsin or endoprotease LysC. Digest aliquots were subjected to nanoLC-ESI-LIT-MS/MS analysis, which was performed with a LTQ XL mass spectrometer (Thermo, USA) connected to an Easy-nanoLC (Thermo) as already reported[1,2]. Database searching with nanoLC-ESI-LIT-MS/MS data was performed as already reported[1,2]; identification results are reported Supplementary Data File 3.

APE interactome analysis

Immunopurified protein from total cell extracts of HeLa cells silenced for APE1 endogenous protein and re-expressing ectopic $APE1^{WT}$ or $APE1^{N\Delta33}$ FLAG-tagged protein and grown under different experimental conditions, or from endogenous APE1-silenced HeLa cells stably transfected with the empty vector and expressing a scrambled siRNA sequence ($APE1^{SCR}$) were analysed in parallel by SDS-PAGE. After colloidal Coomassie staining, whole gel lanes were cut into six slices, minced and washed with water. Corresponding proteins were separately *in-gel* reduced, S-alkylated with iodoacetamide and digested with trypsin, as previously reported[3–5]. Individual protein digests were then analyzed by nLC-ESI-LIT-MS/MS[3–5]. Each sample was analyzed in duplicate under two different MS/MS data acquisition conditions to increase the number of identified peptides/protein coverage. Resulting data were searched with the Proteome Discoverer (version 1.3) software package (Thermo, USA) against a non-redundant human sequence database (NCBI 02/04/2014) as already reported[3–5]. Definitive peptide assignment was always associated with manual spectra visualization and verification. Identification results are reported in Supplementary Data File 4 and 5. Putative interacting partners of $APE1^{WT}$ and $APE1^{N\Delta33}$ (Supplementary Data File 2) were assigned by subtracting data obtained for each gel slice with the corresponding ones from the control sample ($APE1^{SCR}$).

Gene annotations co-occurrence analysis

Gene IDs corresponding to the APE1-interacting proteins identified by proteomic analysis in this study plus the other already known in literature were submitted to GeneCodis (http://genecodis.cnb.csic.es/), a web-based tool for the ontological analysis, selecting *H. sapiens* as

the source for the annotations and Gene Ontology categories to perform the gene annotation co-occurrence analysis.

Co-immunoprecipitation

Co-immunoprecipitation analyses were performed as already described[1]. For DNase and RNase treatments, cell lysates were pretreated with 100 U ml^{-1} DNase I and 100 µg ml^{-1} DNase-free RNase A, for 30 min, at 30 °C, before coimmunoprecipitation[6].

RNA immunoprecipitation

Before harvesting, $APE1^{WT}$ FLAG-tagged expressing cells were washed twice with PBS, harvested by trypsinization and centrifuged at 250 × *g* for 5 min, at 4 °C. Supernatant was removed, and cells were resuspended in 10 ml PBS. Formaldehyde was added to a final concentration of 1% v/v, and cross-linking was performed for 10 min at room temperature. Then, glycine was added to a final concentration of 125 mM to quench cross-linking, and the cells centrifuged again. Cell pellets were washed twice with ice-cold PBS containing 1X protease inhibitor cocktail (Sigma), 0.5 mM phenylmethylsulfonyl fluoride (PMSF), 1 mM NaF, 1 mM Na_3VO_4, and 0.5 U/ml RNaseOUT (Invitrogen), and centrifuged again as described above. Then, cells were lysed in 300 µl of ChIP lysis buffer containing 50 mM Tris-HCl pH 7.4, 150 mM NaCl, 1 mM EDTA and 1% w/v Triton X-100 completed with the same inhibitors quoted above. Lysates were sonicated with a Bioruptor instrument (Diagenode, Liege, Belgium) for three times and on continuous sonication for 30 s. After

sonication, insoluble elements were cleared by microcentrifugation at maximum speed for 10 min, at 4 °C, and supernatants were diluted 10 fold in ChIP Dilution Buffer (0.01% w/v SDS, 1.1% w/v Triton X-100, 1.2 mM EDTA, 16.7 mM Tris-HCl pH 8.1 and 167 mM NaCl) added with protease inhibitors and 0.5 U ml^{-1} RNAseOUT (Invitrogen). Immunoprecipitation was performed by using ANTI-FLAG® M2 Affinity Gel (Sigma). Forty µl of resin per reaction was used, which was added with 0.4 µg of salmon sperm DNA (Sigma) and 1 µg of BSA (Sigma). Samples were then placed in a roller shake overnight and centrifuged at 8000 × *g* for 1 min, at 4 °C. Supernatants were discarded and the resin was washed once with 1 ml of Low Salt Immune Complex Wash Buffer (0.1% w/v SDS, 1% w/v Triton X-100, 2 mM EDTA, 20 mM Tris-HCl pH 8.1 and 150 mM NaCl), High Salt Immune Complex Wash Buffer (0.1% w/v SDS, 1% w/v Triton X-100, 2 mM EDTA, 20 mM Tris-HCl pH 8.1 and 500 mM NaCl), LiCl Immune Complex Wash Buffer (0.25 M LiCl, 1% w/v NP40, 1% w/v deoxycholate, 1 mM EDTA, 10 mM Tris-HCl pH 8.1) and twice with TE buffer (10 mM Tris-HCl, 1 mM EDTA pH 8.0) for 5 min, at room temperature, with rotation. Immune complexes were eluted twice in rotation mixing for 15 min by addition of 250 µl of Elution Buffer (1% w/v SDS and 0.1 M $NaHCO_3$ supplemented with 0.5 U/ml RNaseOUT). NaCl was added to a final concentration of 200 mM then placed at 65°C, for at least 2 h, to reverse cross-linking. Next, the released RNA was digested with 20 µl of 1 M Tris-HCl pH 6.5, 10 µl of 0.5 M EDTA, and 20 µg of Proteinase K (Sigma), at 42 °C, for 45 min. Samples were subjected to RNA extraction using QIAzol reagent (miRNeasy kit, Qiagen, Germany) according to the manufacturer’s instructions supplemented with an additional on-column DNase digestion.

Supplementary References

1. Lirussi, L. *et al.* Nucleolar accumulation of APE1 depends on charged lysine residues that undergo acetylation upon genotoxic stress and modulate its BER activity in cells. *Mol. Biol. Cell* **23,** 4079–4096 (2012).
2. Fantini, D. *et al.* Critical lysine residues within the overlooked N-terminal domain of human APE1 regulate its biological functions. *Nucleic Acids Res.* **38,** 8239–8256 (2010).
3. Buanne, P. *et al.* Characterization of carbonic anhydrase IX interactome reveals proteins assisting its nuclear localization in hypoxic cells. *J. Proteome Res.* **12,** 282–292 (2013).
4. Lucci, V., Di Palma, T., D'Ambrosio, C., Scaloni, A. & Zannini, M. AMOTL2 interaction with TAZ causes the inhibition of surfactant proteins expression in lung cells. *Gene* **529,** 300–306 (2013).
5. Somma, D. *et al.* CIKS/DDX3X interaction controls the stability of the Zc3h12a mRNA induced by IL-17. *J. Immunol. Baltim. Md 1950* **194,** 3286–3294 (2015).
6. Vidal, A. E., Boiteux, S., Hickson, I. D. & Radicella, J. P. XRCC1 coordinates the initial and late stages of DNA abasic site repair through protein-protein interactions. *EMBO J.* **20,** 6530–6539 (2001).
7. Cun, Y. *et al.* Silencing of APE1 enhances sensitivity of human hepatocellular carcinoma cells to radiotherapy in vitro and in a xenograft model. *PloS One* **8,** e55313 (2013).
8. Jobert, L. & Nilsen, H. Regulatory mechanisms of RNA function: emerging roles of DNA repair enzymes. *Cell Mol Life Sci* **71,** 2451-65 (2014).
9. Masuda, Y., Bennett, R. A. & Demple, B. Dynamics of the interaction of human apurinic endonuclease (Ape1) with its substrate and product. *J. Biol. Chem.* **273,** 30352–30359 (1998).

10. Bhakat, K. K., Izumi, T., Yang, S. H., Hazra, T. K. & Mitra, S. Role of acetylated human AP-endonuclease (APE1/Ref-1) in regulation of the parathyroid hormone gene. *EMBO J* **22,** 6299–309 (2003).
11. Choi, Y., Sims, G. E., Murphy, S., Miller, J. R. & Chan, A. P. Predicting the functional effect of amino acid substitutions and indels. *PloS One* **7,** e46688 (2012).
12. Kaina, B. *et al.* BER, MGMT, and MMR in defense against alkylation-induced genotoxicity and apoptosis. *Prog. Nucleic Acid Res. Mol. Biol.* **68,** 41–54 (2001).
13. Kelley, M. R. DNA Repair In Cancer Therapy. Molecular targets and clinical applications. (2012).
14. Kumar, P., Henikoff, S. & Ng, P. C. Predicting the effects of coding non-synonymous variants on protein function using the SIFT algorithm. *Nat. Protoc.* **4,** 1073–1081 (2009).
15. Bennett, R. A., Wilson, D. M., Wong, D. & Demple, B. Interaction of human apurinic endonuclease and DNA polymerase beta in the base excision repair pathway. *Proc. Natl. Acad. Sci. U. S. A.* **94,** 7166–7169 (1997).
16. Condemine, W., Takahashi, Y., Le Bras, M. & de Thé, H. A nucleolar targeting signal in PML-I addresses PML to nucleolar caps in stressed or senescent cells. *J. Cell Sci.* **120,** 3219–3227 (2007).
17. Adzhubei, I. A. *et al.* A method and server for predicting damaging missense mutations. *Nat. Methods* **7,** 248–249 (2010).
18. Percipalle, P. & Louvet, E. In vivo run-on assays to monitor nascent precursor RNA transcripts. *Methods Mol Biol* **809,** 519–33 (2012).
19. Adelmant, G. *et al.* DNA ends alter the molecular composition and localization of Ku multicomponent complexes. *Mol. Cell. Proteomics MCP* **11,** 411–421 (2012).

20. Vascotto, C. *et al.* APE1/Ref-1 interacts with NPM1 within nucleoli and plays a role in the rRNA quality control process. *Mol Cell Biol* **29,** 1834–54 (2009).

21. Rommer, A. *et al.* Overexpression of primary microRNA 221/222 in acute myeloid leukemia. *BMC Cancer* **13,** 364 (2013).

22. FANTOM Consortium and the RIKEN PMI and CLST (DGT) *et al.* A promoter-level mammalian expression atlas. *Nature* **507,** 462–470 (2014).

23. Weibrecht, I. *et al.* Proximity ligation assays: a recent addition to the proteomics toolbox. *Expert Rev Proteomics* **7,** 401–9 (2010).
