# Supplementary material for: Mammalian APE1 controls miRNA processing and its interactome is linked to cancer RNA metabolism
Source: Nat Commun. 2017 Oct 6;8:797. doi: 10.1038/s41467-017-00842-8 (PMC5630600; doi:10.1038/s41467-017-00842-8)
Supplement: Supplementary file 3 — Description of Additional Supplementary Files [file 41467_2017_842_MOESM3_ESM.pdf]

Description of Additional Supplementary Files

File Name: Supplementary Data 1
Description: Sheet A. List of differentially expressed miRNAs profiled in H_2O_2-treated and APE1-knocked down HeLa cells (n=30 and n=55, respectively). Only transcripts significantly up-regulated (green cells) or down-regulated (red cells) are shown (≥1 logFC, q-value≤0.1). Values are ordered by decreasing logFC. Sheet B. Overall miRNA expression profiling (n=800) of HeLa scramble clones (SCR) non-treated and treated with 1 mM H_20_2 for 15 min. Values are ordered by increasing qvalue. Sheet C. Overall miRNA expression profiling (n=800) of HeLa scramble clones (SCR) non-treated and subjected to APE1-knocked down. Values are ordered by increasing qvalue. ID: miRNA Identifier; logFC: log2-normalized expression fold change; lr: likelihood ratio test statistics; pvalue: p-value; qvalue: adjusted p-value (Benjamini and Hochberg procedure). Sheet D. The canonical pathways associated with the target genes of miRNAs modulated after APE1 silencing. Statistical significance, ratio of genes included in the pathway and the name of the genes are shown. Target genes prediction and pathway enrichment analysis were performed using Ingenuity Pathway Analysis. Sheet E. Starting from the mirTarbase database, we retrieved the validated target gene list for each miRNA. Then, we managed the data in order to obtain the list of all miRNA able to target each gene.

File Name: Supplementary Data 2
Description: APE1-interacting partners as revealed in this study by combined SDS-PAGE/trypsinolysis/nanoLCESI-LIT-MS/MS analysis or as determined by our group in a previous interactome investigation based on two-dimensional electrophoresis and MALDI-TOF peptide fingerprinting analysis[20]. Protein name, SwissProt accession number, molecular mass (kDa), known protein functions and slice number in Supplementary Fig. 4b and 4c are listed. Identification details are reported in Supplementary Table 3 and Table 4. Protein ability to interact with the $APE1^{N33}$ or with $APE1^{WT}$ after TSA treatment is also reported. The down arrows indicate a decreased interaction with respect to $APE1^{WT}$. Proteins previously identified by us as APE1-interacting partners are indicated in italics.

File Name: Supplementary Data 3
Description: Acetylated peptides identified in the tryptic digest of $APE1^{WT}$. Peptides with modified residue(s) assigned with certainty or uncertainty (for position) are highlighted in white and grey background, respectively. Modified peptide, modified residue(s), peptide sequence and modificable residues (k) therein, peptide identification scores (Mascot ion score and Sequest X_{corr} value), peptide charge state and MH^+ values are shown. Identification details are reported in Supplementary Table 4.

File Name: Supplementary Data 4
Description: Identification details of acetylated peptides identified in the tryptic digest of $APE1^{WT}$. Non-modified and modified tryptic peptides, modified residue(s), peptide sequence, modifiable residues (k), peptide identification scores (Mascot ion score and Sequest X_{corr}

value), peptide charge state, MH^+ values, DM and matched ions are shown, together with other analytical parameters.

File Name: Supplementary Data 5
Description: Summary of the identification data determined for APE1-interacting proteins, as ascertained in this study.

File Name: Supplementary Data 6
Description: Sheet A. List of APE1 interactors. Annotation was performed using IPA. Gene symbols, functional class, gene names, cellular location, biomarkers usage, related drugs, and entrez ID in human, mouse and rat are shown. APE1-interacting partners as revealed in this study by combined SDSPAGE/trypsinolysis/nanoLC-ESI-LIT-MS/MS analysis or as determined by our group in a previous interactomic investigation based on two-dimensional electrophoresis and MALDI-TOF peptide fingerprinting are indicated in bold. Sheet B. Starting from the gene list of the APE1-protein interactome, Ingenuity Pathways analysis was performed, using IPA software version 2016. The resulting annotated biofunctions ('Functions Annotation') were ranked, depending on the number of significantly expressed genes, and were ordered by statistical significance (p-value). Correction for Multiple tests applied only marginal differences to p-values. Sheet C. Complete data reporting all the significant functional enrichments obtained performing GeneCodis analysis. 'GO term ID' and 'Annotations' columns represent the Gene Ontology codes of annotations and the textual description of annotations, respectively. The third and the fourth columns represent the number of genes in the input list and the reference list for a given annotation, respectively. The fifth column represents the total number of genes in the reference list. P-values calculated using hypergeometric distribution and its correction using the stimulation-based approach are reported. The 'Genes' column identifies the set of genes in the input list showing a given annotation.

File Name: Supplementary Data 7
Description: Sheet A. List of expressed features (n=1015) differentially expressed in both IP-APE1 (APEX1) vs Input comparison (fold change >2, fdr2). Sheet B. Subset of the non-coding transcripts differentially expressed in both IP-APE1 vs Input comparison (fold change >2, fdr2). Ensembl transcript ID, Gene Symbol, and HGNC transcript ID are transcript Identifiers; logFC: log2-normalized expression fold change; logCPM: average log2 counts per million; PValue: pvalue; FDR: adjusted p-value (false discovery rate procedure). Sheet C. Starting from the gene list (sheet A), Ingenuity Pathways analysis was performed, using IPA software version 2016. The resulting annotations related to biofunctions ('Functions Annotation') were ranked, depending on the number of significantly expressed genes, and were ordered by the probability of finding the observed molecules enriched in a given annotation by chance (p-Value). Sheet D. Starting from the gene list of sheet A, Ingenuity Pathways analysis was performed, using IPA software version 2016. The resulting annotations related to diseases ('Diseases Annotation') were ranked, depending on the number of significantly expressed genes, and were ordered by the probability of finding the observed molecules enriched in a given annotation by chance (p-Value). Sheet E. Starting

from the gene list of sheet A, DAVID enrichment analysis was performed. For each Enriched functional term (‘Term’), the specific Category, the count of elements, p-value and adjusted p-values are reported.
