# Supplementary material for: Mammalian APE1 controls miRNA processing and its interactome is linked to cancer RNA metabolism
Source: Nat Commun. 2017 Oct 6;8:797. doi: 10.1038/s41467-017-00842-8 (PMC5630600; doi:10.1038/s41467-017-00842-8)
Supplement: Supplementary file 5 — Supplementary Data 2 [file 41467_2017_842_MOESM5_ESM.docx]

| **Protein name** | **SwissProt entry** | **Mr**  **(kDa)** | **Function** | **Interaction with APE1^NΔ33^** | **TSA treatment** | **Slice n.** |
| --- | --- | --- | --- | --- | --- | --- |
| *40S ribosomal protein SA (RSSA)* | *P08865* | *33* | *Required for the assembly and/or stability of the 40S ribosomal subunit. Processes the 20S rRNA-precursor to mature 18S rRNA* | *↓* |  |  |
| *60S acidic ribosomal protein P0 (RLA0)* | *P05388* | *34* | *Functional equivalent of E.coli protein L10* | *No* |  |  |
| 60S ribosomal protein L14 (RL14) | P50914 | 23 | Component of the large subunit of cytoplasmic ribosomes |  | + | T1 |
| 60S ribosomal protein L3 (RL3) | P39023 | 46 | Component of the large subunit of cytoplasmic ribosomes |  | - | A6 |
| 60S ribosomal protein L4 (RL4) | P36578 | 48 | Component of the large subunit of cytoplasmic ribosomes | No |  | A3, A4, A5, T5, T6 |
| Alpha-actinin-1 (ACTN1) | P12814 | 103 | F-actin cross-linking protein |  | - | A3 |
| Aminopeptidase N (APN) | P15144 | 109 | Broad specificity aminopeptidase. Used as a marker for acute myeloid leukemia and played a role in tumor invasion |  |  | A2, T2 |
| Brain acid soluble protein 1 (BASP1) | P80723 | 23 | Trascriptional corepressor and promoter binding protein | Yes |  | A3, A4, A5, N4, N5, T5 |
| Heterogeneous nuclear ribonucleoprotein F (hnRNP-F) | P52597 | 46 | Component of the heterogeneous nuclear ribonucleoprotein (hnRNP) complexes |  |  | A6, T6 |
| Heterogeneous nuclear ribonucleoprotein H (hnRNP-H) | P31943 | 49 | Component of the heterogeneous nuclear ribonucleoprotein (hnRNP) complexes |  | + | T5 |
| Heterogeneous nuclear ribonucleoprotein U (hnRNP-U) | Q00839 | 90 | Binds to pre-mRNA and to to double- and single-stranded DNA and RNA |  | + | T2 |
| *Keratin, type II cytoskeletal 8 (K2C8)* | *P05787* | *50* | *Helps to link contractile apparatus to dystrophin at costameres of striated muscle. Belongs to ribosomal protein S2P family* | *No* |  |  |
| Kinesin-like protein (KIF11) | P52732 | 119 | Motor protein | No |  | A1, A2, T1, T2 |
| Moesin (MOES) | P26038 | 68 | Connects major cytoskeletal structures to the plasma membrane | No |  | A4 |
| Myosin-9 (MYH9) | P35579 | 227 | Role in cytokinesis, cell shape, and specialized functions |  | + | T1 |
| Myosin-Ic (Myosin I beta) (MYO1C) | O00159 | 118 | Unconventional myosins involved in intracellular movements ans Involved in glucose transporter recycling in response to insulin |  | + | T2 |
| Nucleolin (Protein C23) (NCL) | P19338 | 77 | Role in pre-rRNA transcription and ribosome assembly. Chromatin decondensation inducing protein | No |  | A2, A3, A4, T2, T3 |
| *Nucleophosmin (NPM1)* | *P06748* | *35* | *Associated with nucleolar ribonucleoprotein structures and binding to single-stranded nucleic acids; assembly and transport of ribosome* | *No* |  |  |
| *Peroxiredoxin 6 (PRDX6)* | *P30041* | *25* | *Involved in redox regulation of cell and protection against oxidative injury (lipid peroxidation)* | *↓* |  |  |
| Polyadenylate-binding protein 1-A (PABP 1) | P11940 | 71 | Binds the poly(A) tail of mRNA |  | - | A4 |
| *Pre-mRNA-processing factor (PRP19)* | *Q9UMS4* | *53* | *DNA double strand break repair and pre-mRNA splicing reaction* | *No* |  |  |
| *Ribose-phosphate pyrophosphokinase 1 (PRPS1)* | *P60891* | *31* | *Ribose metabolism* | *Yes* |  |  |
| *Ribose-phosphate pyrophosphokinase 2 (PRPS2)* | *P11908* | *31* | *Ribose metabolism* | *Yes* |  |  |
| Spectrin beta chain, brain 1 (SPTB2) | Q01082 | 275 | Involved in secretion, interacts with calmodulin in a calcium-dependent manner |  | + | T1 |
| Splicing factor, proline- and glutamine-rich (SFPQ) | P23246 | 76 | DNA and RNA binding protein; pre-mRNA splicing factor. Involved in NHEJ and required for double strand break. Trascriptional regulator | No |  | A3 |
| *T-complex protein 1 subunit alpha (TCPA)* | *P17987* | *57* | *Molecular chaperone* | *No* |  |  |
| Thyroid hormone receptor-associated protein 3 (THRAP3) | Q9Y2W1 | 109 | Role in transcriptional coactivation | No |  | A2 |
| Y-box-binding protein (YB1) | P67809 | 36 | Mediates pre-mRNA alternative splicing regulation. Binds and stabilizes cytoplasmic mRNA | No |  | A6, T6 |

**Supplementary Table S2. APE1-interacting partners as revealed in this study by combined SDS-PAGE/trypsinolysis/nanoLC-ESI-LIT-MS/MS analysis or as determined by our group in a previous interactome investigation based on two-dimensional electrophoresis and MALDI-TOF peptide fingerprinting.** Protein name, SwissProt accession number, molecular mass (kDa), known protein functions and slice number in Supplementary Fig. 4b and 4c are listed. Identification details are reported in Supplementary Table 5. Protein ability to interact with the APE1^NΔ33^ or with APE1^WT^ after TSA treatment is also reported. The down arrows indicate a decreased interaction with respect to APE1^WT^. Proteins previously identified by us as APE1-interacting partners are indicated in italics.
