# Supplementary material for: Mammalian APE1 controls miRNA processing and its interactome is linked to cancer RNA metabolism
Source: Nat Commun. 2017 Oct 6;8:797. doi: 10.1038/s41467-017-00842-8 (PMC5630600; doi:10.1038/s41467-017-00842-8)
Supplement: Supplementary file 6 — Supplementary Data 3 [file 41467_2017_842_MOESM6_ESM.docx]

**Supplementary Table 3. Acetylated peptides identified in the tryptic digest of APE1^WT^.** Peptides with modified residue(s) assigned with certainty or uncertainty (for position) are highlighted in white and grey background, respectively. Modified peptide, modified residue(s), peptide sequence and modificable residues (k) therein, peptide identification scores (Mascot ion score and Sequest X_corr_ value), peptide charge state and MH^+^ values are shown. Identification details are reported in Supplementary Information Table S4.

| **Peptide** | **Modified residue(s)** | **Peptide sequence and modificable residue(s)** | **Mascot ion score** | **Sequest X_corr_** | **Charge** | **MH^+^ (Da)** |
| --- | --- | --- | --- | --- | --- | --- |
| (25-52)Ac | K25, K27, K31, K32 or K35 | kSkTAAkkNDkEAAGEGPALYEDPPDHK | 26, 36, 37, 38 |  | 4 | 3039.9 |
| (28-52)Ac | K31, K32 or K35 | TAAkkNDkEAAGEGPALYEDPPDHK | 39, 40, 41, 42, 43 |  | 3 | 2693.9 |
| (32-52)Ac | K32 or K35 | kNDkEAAGEGPALYEDPPDHK |  | 2.30, 2.31 | 3 | 2322.1 |
| (137-156)Ac | K141 | QcPLkVSYGIGDEEHDQEGR | 56 | 4.76 | 3 | 2358.6 |
| (194-221)Ac_2_ | K194, K197 or K194, K203 or K197, K203 | kFLkGLASRkPLVLcGDLNVAHEEIDLR | 20 | 2.27, 2.29, 2.63 | 3 | 3275.1 |
| (195-221)Ac | K197 | FLkGLASRkPLVLcGDLNVAHEEIDLR | 33 |  | 4 | 3106.4 |
| (195-221)Ac_2_ | K197 and K203 | FLkGLASRkPLVLcGDLNVAHEEIDLR | 23 |  | 3 | 3148.3 |
| (222-254)Ac_2_ | K224, K227 or K224, K228 or K227, K228 | NPkGNkkNAGFTPQERQGFGELLQAVPLADSFR | 21, 31 |  | 4 | 3696.5 |
| (225-237)Ac | K228 | GNKkNAGFTPQER |  | 2.93 | 3 | 1489.1 |
| (225-237)Ac_2_ | K227 and K228 | GNkkNAGFTPQER | 22 |  | 3 | 1529.8 |
| (228-237)Ac | K228 | kNAGFTPQER | 23 |  | 2 | 1189.6 |
